# Mitogenomes from type specimens, a genotyping tool for morphologically simple species: ten genomes of agar-producing red algae

**DOI:** 10.1038/srep35337

**Published:** 2016-10-14

**Authors:** Ga Hun Boo, Jeffery R. Hughey, Kathy Ann Miller, Sung Min Boo

**Affiliations:** 1 University Herbarium, University California, 1001 Valley Life Sciences Building #2465, Berkeley, CA 94720, USA; 2 Department of Biology, Chungnam National University, Daejeon 305-764, Korea; 3 Division of Mathematics, Science, and Engineering, Hartnell College, 411 Central Ave., Salinas, CA 93901, USA

## Abstract

DNA sequences from type specimens provide independent, objective characters that enhance the value of type specimens and permit the correct application of species names to phylogenetic clades and specimens. We provide mitochondrial genomes (mitogenomes) from archival type specimens of ten species in agar-producing red algal genera *Gelidium* and *Pterocladiella*. The genomes contain 43–44 genes, ranging in size from 24,910 to 24,970 bp with highly conserved gene synteny. Low Ka/Ks ratios of apocytochrome b and cytochrome oxidase genes support their utility as markers. Phylogenies of mitogenomes and *cox*1+*rbc*L sequences clarified classification at the genus and species levels. Three species formerly in *Gelidium* and *Pterocladia* are transferred to *Pterocladiella*: *P*. *media* comb. nov., *P*. *musciformis* comb. nov., and *P*. *luxurians* comb. and stat. nov. *Gelidium sinicola* is merged with *G*. *coulteri* because they share identical *cox*1 and *rbc*L sequences. We describe a new species, *Gelidium millariana* sp. nov., previously identified as *G*. *isabelae* from Australia. We demonstrate that mitogenomes from type specimens provide a new tool for typifying species in the Gelidiales and that there is an urgent need for analyzing mitogenomes from type specimens of red algae and other morphologically simple organisms for insight into their nomenclature, taxonomy and evolution.

Voucher specimens of algae, fungi and plants housed in 3,400 public herbaria are the basis for systematic and evolutionary studies^1^. Type specimens are particularly valuable because they represent the material on which species descriptions are based, and because the scientific name is anchored to the type specimen. Morphological comparisons of new collections with type material are entirely or partly subjective, while molecular sequences from type specimens provide independent, objective characters that enhance the value of type specimens and permit the application of correct names to phylogenetic clades and specimens^2^.

Although damage to DNA occurs during specimen preparation and long-term herbarium storage, sequences from archival specimens can provide reliable data for taxonomic and evolutionary studies^3^. DNA sequence data have demonstrated that many species of red algae (Rhodophyta) are complexes of cryptic evolutionary lineages that cannot be recognized by comparison of morphological characters alone^4^,^5^,^6^,^7^,^8^. Sequences from type specimens clarify not only the number of taxa within such complexes but also provide names for those that have been previously described. Similarly, species with simple and environmentally variable morphologies, especially ones for which reproductive individuals are rare and thus lack diagnostic morphological and anatomical characters, are difficult to distinguish at the genus and species level. In these cases, sequence information from type specimens is the gold standard for species identification^6^,^9^,^10^.

Methods for extracting and sequencing DNA from type specimens have been developed for taxonomic studies of red algae^2^,^4^,^5^. Saunders & McDevit^11^ reported that DNA analysis of archival specimens is not practical, due to the very low rate of successful amplifications and high levels of DNA contamination. In their commentary paper, Hughey & Gabrielson^12^ provided detailed laboratory protocols for the successful extraction of archival DNA. However, phylogenetic information recovered from archival specimens is typically limited due to the short length of sequences (about 200 base pairs [bp] or less) and to *rbc*L, a conserved marker^10^,^13^,^14^,^15^. Next-generation sequencing (NGS) is a cost-effective technique to derive sequence information, including complete organellar genomes, from the many valuable specimens stored in herbaria around the world^16^. Hughey *et al*.^2^ illustrated this by analyzing the complete mitochondrial and plastid genomes from nine type specimens representing three *Pyropia* species (Bangiales, Bangiophyceae, Rhodophyta).

Gelidioid red algae (Gelidiales, Florideophyceae) are the most significant marine source for agar and agarose industries. Interest in these marine resources is intensifying because they are in short supply globally and the price of agar has tripled on the world market^17^. Since the first molecular work by Freshwater & Rueness^18^, analyses using *rbc*L gene sequences and other markers have resolved some of the genus- and species-level taxonomic problems in the Gelidiales^8^,^19^,^20^,^21^,^22^. Multiple short sequences of *rbc*L from type specimens, generated by using nested primers, have been patched together to produce longer, more useful sequences (400 to 1,200 bp). For example, sequences from lectotype material of *Gelidiella tenuissima* Feldmann & Hamel (type locality: Biarritz, France) enabled the identification of specimens from Gran Canaria^7^. Grusz & Freshwater^13^ analyzed sequences from the isotype of *Gelidium sclerophyllum* W.R.Taylor (type locality: Esmeraldas, Ecuador) and compared them with contemporary specimens from Costa Rica. Iha *et al*.^14^ worked with sequences from the holotype of *Gelidium coarctatum* Kütz. (type locality: Pernambuco, Brazil) to reveal a misidentification, i.e., *G*. *coarctatum* = *G*. *capense* (S.G.Gmel.) P.C.Silva, and enable the description of a new species in Brazil. These studies demonstrate that sequence information from type specimens in the Gelidiales is useful for species identification. For phylogenetic studies, complete sequences of mitochondrial and plastid genes, including *rbc*L, are available with NGS, as we demonstrate here.

Recently, a five-gene phylogeny, including nuclear *Ces*A, has improved the taxonomic resolution of this order with the proposal of a new family, the Orthogonacladiaceae, and a new genus, *Orthogonacladia*^23^. However, many challenging questions at the genus and species level remain. For example, *Gelidium crinale* f. *luxurians* Collins was merged with *Pterocladia media* E.Y.Dawson^24^; it has been suggested that this species may belong in the genus *Pterocladiella*^25^,^26^. Reports of *G*. *isabelae* W.R.Taylor in Australia and South Africa^27^ and *G*. *galapagense* W.R.Taylor in Korea^28^, far from their type localities in the Galápagos Islands, require critical examination.

Thirty-eight mitogenomes from red algae have been analyzed^29^,^30^. In the Gelidiales, mitogenomes have been constructed for *Gelidium elegans* Kütz. and *G*. *vagum* Okamura^29^,^31^. However, because most red algal mitogenomes are highly conserved, as in other organisms^32^, investigations into their contribution to phylogenetic studies have been limited. Here we used NGS to generate mitogenomes from ten archival type specimens (eight holotypes and two isotypes) housed in the University Herbarium, University California at Berkeley (UC). Our goals are: i) to provide an objective tool for identifying species by generating mitogenomes from type specimens, ranging from 49 to 118 years old, of species in the Gelidiales, ii) to assess the values of mitochondrial protein-coding genes as markers to improve our understanding of taxonomy and evolution, iii) to re-examine the generic status of species in *Gelidium* and *Pterocladia*, and iv) to describe a new species.

## Results

### Gelidiales mitogenomes

**Figure 1:**
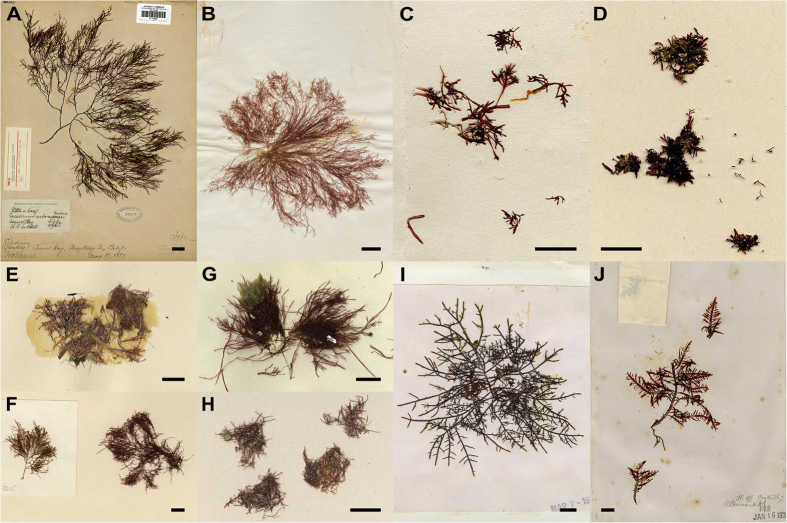
The ten archival type specimens of the Gelidiales from the University Herbarium, University of California at Berkeley (UC). (**A**) *Gelidium arborescens*, UC93582, holotype; (**B**) *Gelidium crinale* f. *luxurians*, UC1878479, holotype; (**C**) *Gelidium galapagense*, UC1884224, holotype; (**D**) *Gelidium isabelae*, UC1884226, holotype; (**E**) *Gelidium sclerophyllum*, UC1884229, holotype; (**F**) *Gelidium sinicola*, UC276620, holotype; (**G**) *Pterocladia media*, UC1884019, isotype; (**H**) *Pterocladia musciformis*, UC1884021, holotype; (**I**) *Pterocladia mexicana*, UC694722, isotype; (**J**) *Pterocladia robusta*, UC1884024, holotype. Scale bars: A, 2 cm; B–J, 1 cm.

Mitogenomes from ten archival type specimens in the Gelidiales (Fig. 1; Supplementary Table S1) were constructed using NGS methodologies. The mitogenomes consisted of 43 to 44 genes, with lengths ranging from 24,910 bp in *Gelidium crinale* f. *luxurians* to 24,970 bp in *Gelidium galapagense* (Supplementary Table S2). The GC content ranged from 28.1% in *Pterocladia musciformis* W.R.Taylor to 30.2% in *G. sinicola* N.L.Gardner (Supplementary Table S2). The mitogenomes contained 23 protein-coding genes, 18–19 tRNAs, and 2 rRNA subunits; they are similar to the published mitogenomes of *G. elegans* and *G*. *vagum*, which we included in our analyses (Supplementary Table S2; Figs S1–S10).

A total of 19 tRNA coding genes was detected, the anticodon sequences of which are shown in Supplementary Table S3. The sole difference among species was that Trn-His-(GTG) was found in *G*. *arborescens*, *G*. *elegans*, *G*. *sclerophyllum* W.R.Taylor, *Pterocladia media*, and *P*. *musciformis*, whereas trn-Gly-(TCC) was found in *G*. *crinale* f. *luxurians*, *P*. *robusta* and *P*. *mexicana*.

**Figure 2:**
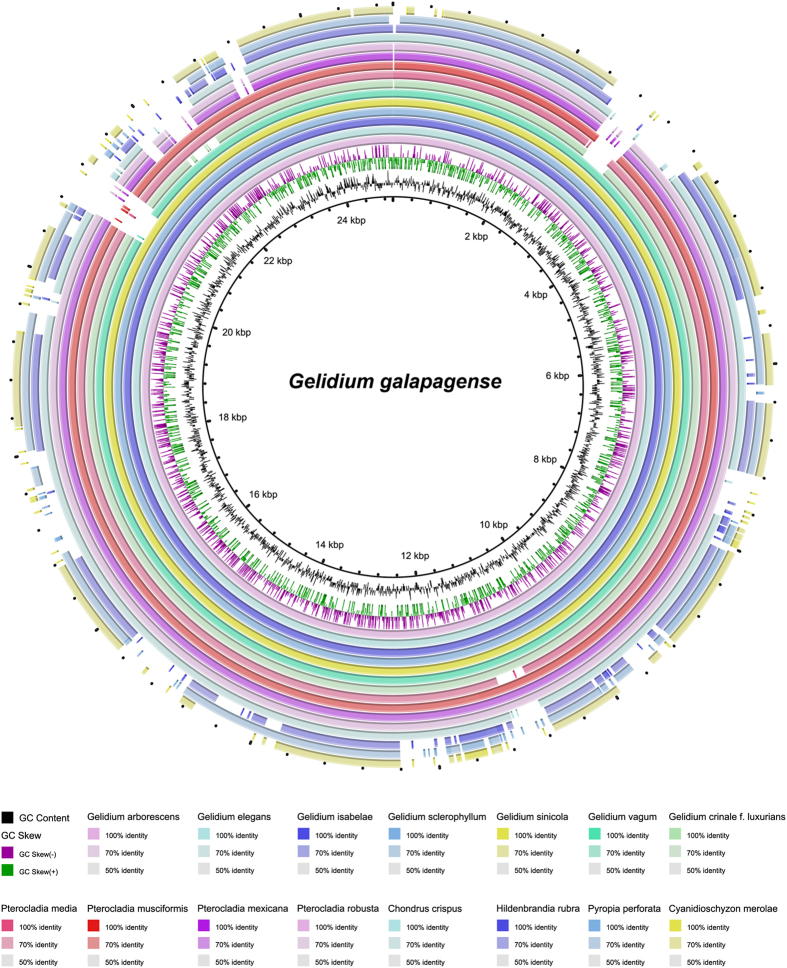
Mitogenome sequence comparison generated using BRIG with *Gelidium galapagense* as reference. The concentric rings represent the sequences of each of the red algal mitogenomes compared with the reference. From this map, the four outermost rings, corresponding to *Cyanidioschyzon merolae*, *Pyropia perforata*, *Hildenbrandia rubra*, and *Chondrus crispus*, have more incongruent regions as indicated by the wider gaps in the rings. The intensity of the ring color denotes the degree of sequence conservation at that region. Gaps or white spaces indicate highly variable regions (<50% sequence similarity).

**Figure 3:**
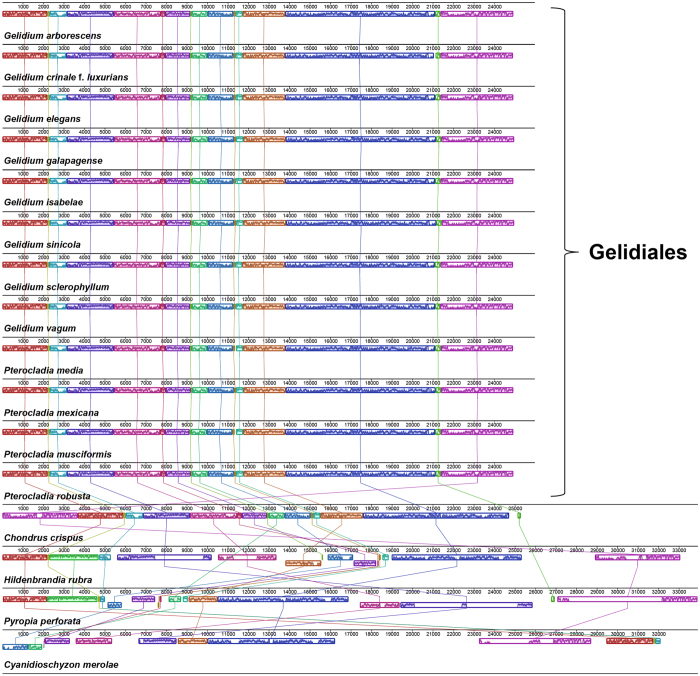
Mauve genome alignments of Gelidiales. Corresponding colored boxes indicate locally collinear blocks (LCBs), which represent homologous gene clusters.

Mitogenome organizations in the Gelidiales are very consistent, lacking rearrangements of protein-coding genes (Figs 2 and 3). The mitogenome sequences of *Cyanidioschyzon*, *Pyropia*, *Hildenbrandia*, and *Chondrus* species are far more incongruent as seen in the larger gap regions in the four outermost rings (Fig. 2). The sequences of red algal mitogenomes were fairly conserved, with about 70% sequence identity in most regions of the genome relative to that of *G*. *galapagense* (Fig. 2).

Interspecific pairwise divergences in *Gelidium* ranged from 2.2–10.8% in *atp*9 to 12.9–32.5% in *sec*Y (Supplementary Table S4). Pairwise divergences in *Pterocladia* were also lower in *atp*9 (3.5–9.1%), and higher in *sec*Y (16.5–35.0%). Between *P*. *mexicana* and *P*. *robusta*, the pairwise divergences were from 0% in *ymf*39 to 1.1% in *nad*3.

**Figure 4:**
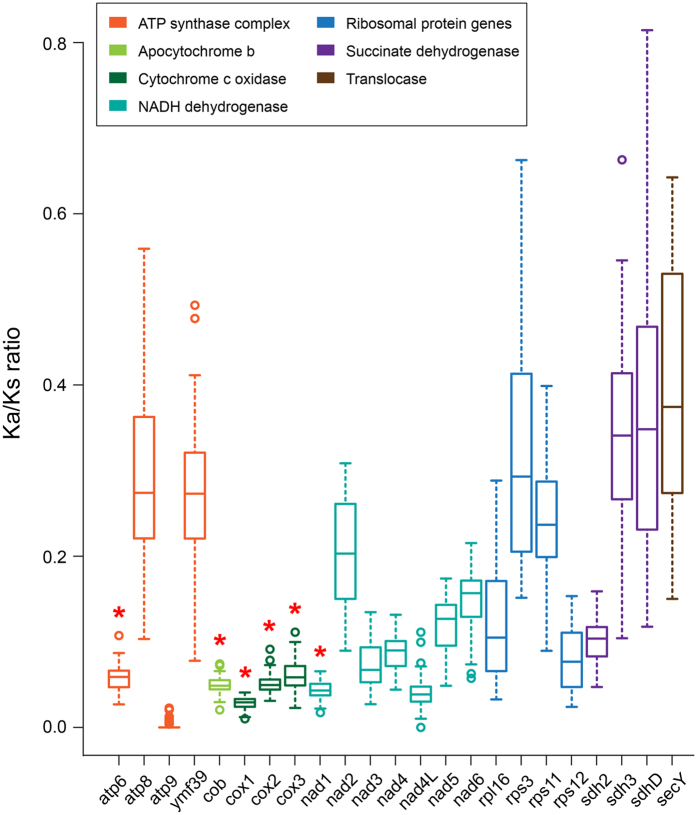
A comparison of the Ka/Ks ratio of 23 protein-coding genes in the Gelidiales. The box color indicates functional groups: ATP synthase complex, apocytochrome b, cytochrome c oxidase, NADH dehydrogenase, ribosomal protein genes, succinate dehydrogenase, and translocase. Asterisks indicate suitable markers for phylogenetic and taxonomic studies.

The mean value of the ratio of nonsynonymous (Ka) versus synonymous substitutions (Ks) for 23 protein-coding genes was in a range of 0.0022–0.3943. The values were less than 0.1 for eleven gene (0.0278 for *cox*1), and more than 0.1 for twelve genes. *Sdh*D (0.3621) and *sec*Y (0.3943) showed notably higher Ka/Ks ratios (Fig. 4; Supplementary Table S4).

### Molecular phylogeny of the Gelidiales

The mitogenome phylogeny, based on twenty-three protein-coding genes, showed that all mitogenomes were distinct except for those of *Pterocladia mexicana* W.R.Taylor and *P*. *robusta* W.R.Taylor, which had low pairwise divergences (0.5%). Five species were included in the *Gelidium* clade (100/1.0 [ML/BPP]); however, *G*. *crinale* f. *luxurians* was nested in *Pterocladia* (100/1.0) (Supplementary Fig. S11). Separation of *Gelidium* from *Pterocladia* was supported by individual protein-coding gene phylogenies (Supplementary Fig. S12)

**Figure 5:**
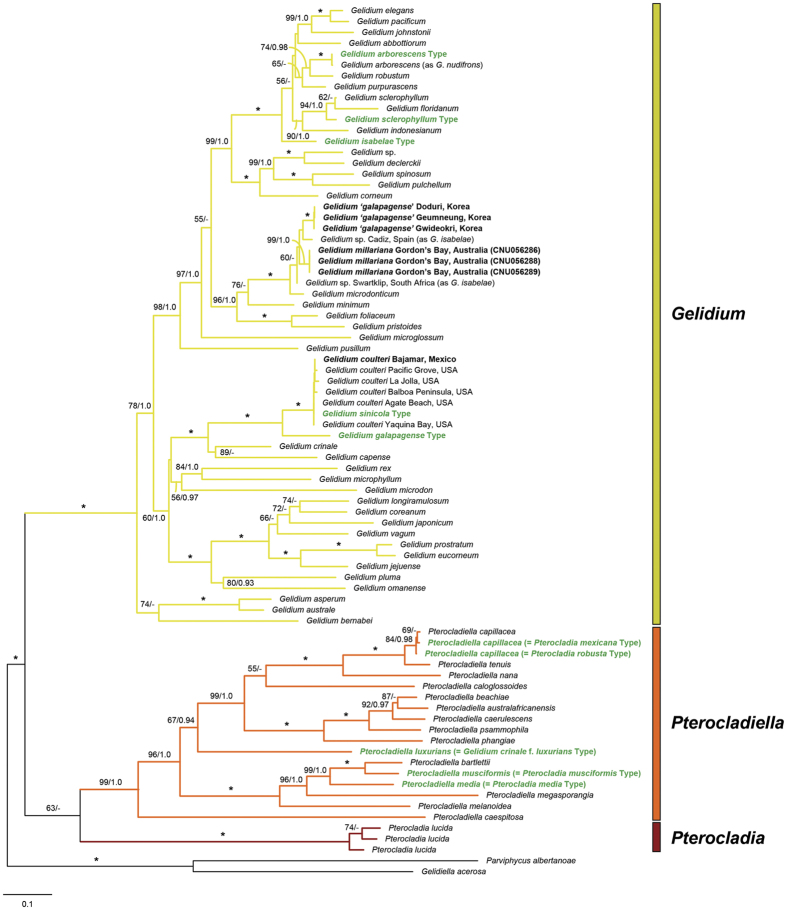
Maximum likelihood tree of *cox*1 + *rbc*L sequences from *Gelidium*, *Pterocladia* and *Pterocladiella* using the GTR + G + I model. Statistically supported bootstrap values (≥50%) and Bayesian posterior probabilities (≥0.90) are shown. Asterisks indicate full support in both analyses. The bold green color indicates type sequences and bold black color indicates contemporary, freshly collected specimens.

The concatenated (*cox*1 + *rbc*L) phylogeny derived from sequences representing 80 taxa including two outgroups was highly concordant with the individual gene phylogenies (Fig. 5; Supplementary Figs S13, S14). *Gelidium arborescens*, *G. galapagense*, *G. isabelae*, *G. sclerophyllum*, and *G. sinicola* were resolved in the clade containing *Gelidium* species (100/1.0). Comparison of *cox*1 sequences from the type specimens with those from contemporary specimens showed that *G. arborescens* was identical to a contemporary specimen identified as *G*. *nudifrons* N.L.Gardner. *Gelidium sinicola* was in a clade containing only *G*. *coulteri* sequences.

*Gelidium isabelae* from Australia was distantly related to the type specimen of *G. isabelae*. Korean *G. galapagense* was also distantly related to the type specimens of *G. galapagense*. Australian *G. isabelae* was more closely related to Korean *G. galapagense*, but the pairwise divergences between these two taxa were 3.0–3.1% (36–37 bp).

The *Pterocladiella* clade was strongly supported (99/1.0; Fig. 5), and included *Gelidium crinale* f. *luxurians*, *Pterocladia media*, *P*. *mexicana*, *P*. *musciformis*, and *P*. *robusta*. Sequences from the type specimen of *Pterocladia mexicana* were similar to those from *P*. *robusta*, with pairwise divergences of 0.4% in *cox*1 and 0.1% in *rbc*L, and both were similar to sequences of *Pterocladiella capillacea* (pairwise divergences, 0.4–0.5% in *cox*1 and 0.1–0.3% in *rbc*L).

### Taxonomic results

On the basis of sequences from type specimens and from fresh collections, we i) describe a new species, *Gelidium millariana* sp. nov. based on specimens identified as *G*. *isabelae* in Australia, ii) transfer three species of *Gelidium* and *Pterocladia* to *Pterocladiella*, and iii) merge *Gelidium sinicola* with *G*. *coulteri*, according to the International Code of Nomenclature for algae, fungi, and plants^33^.

**Figure 6:**
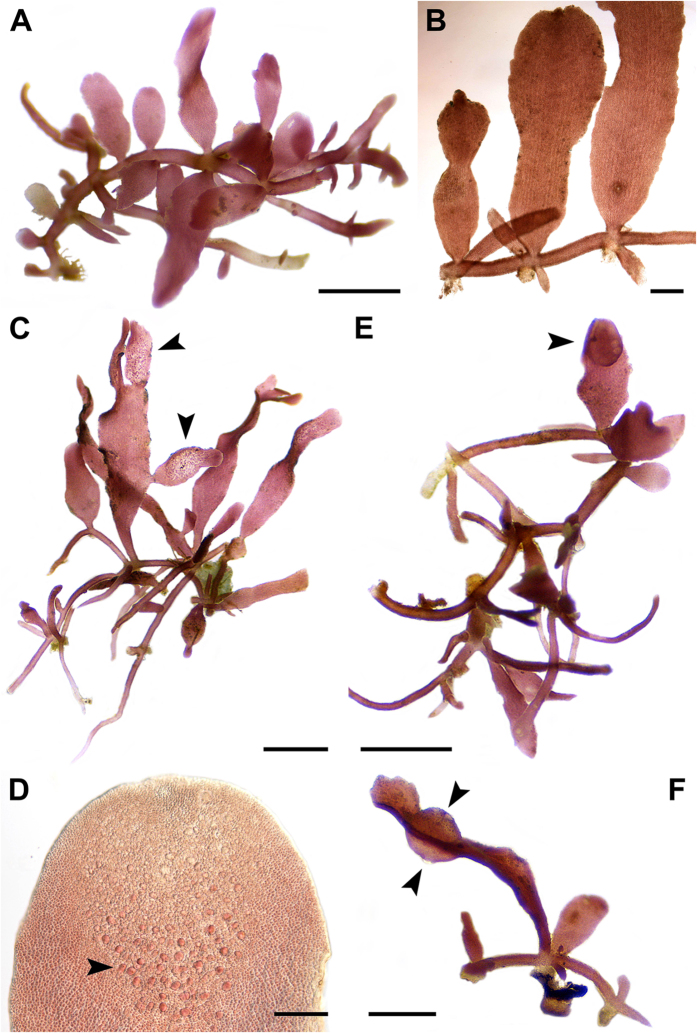
*Gelidium millariana* G.H.Boo, Hughey, K.A.Mill. & S.M.Boo sp. nov. (**A**) Habit of a plant with lanceolate branches on prostrate axes, CNU056289; (**B**) Enlarged view of erect branches, CNU056289; (**C**) Plant with tetrasporangial sori (arrowheads), CNU056286; (**D**) Tetrasporangial sorus showing irregularly arranged tetrasporangia (arrowhead), CNU056286; (**E**) Plant with a cystocarp on erect branch (arrowhead), CNU056288; (**F**) Side view of a cystocarp with openings on both sides (arrowheads), CNU056288. Scale bars: A, C, E, 1 mm; B, D, 100 μm; F, 0.5 mm.

***Gelidium millariana***G.H.Boo, Hughey, K.A.Mill. & S.M.Boo **sp. nov.** (Fig. 6)

Description: Plant (Fig. 6A) light to dark red, up to 1 cm high, forming turf on intertidal rocks; composed of terete prostrate axes with brush-like haptera. Branches lanceolate to clavate, flattened; up to three orders of distichous branching; erect branches cylindrical to compressed at base, becoming flattened distally, 0.3–0.8 mm in width (Fig. 6B). Apices mostly obtuse with prominent apical cell; cortex consisting of 3–5 layers of globose to elliptical cells in cylindrical to compressed sections of erect branches, 2–3 layers in flattened sections; medulla narrow in flattened branches, consisting of elongated thick-walled cells with narrow lumens; rhizines abundant in inner cortex and medulla. Tetrasporangial sori on secondary branches with a sterile margin (Fig. 6C); tetrasporangia irregularly arranged (Fig. 6D), up to 28 μm in diameter. Cystocarps on upper branches, spherical to ovoid, with openings to both surfaces of branches (Figs 6E, 6F). Male plants not observed.

Type: NSW 614432 (image in Fig. 75, Miller & Freshwater 2005^27^), Neds Beach, outer intertidal rock, NSW, Australia, 1.x.2002, *D.W. Freshwater & A.J.K. Millar*; isotypes NSW 614435, WNC2003002-B.

Etymology: The name honors Dr. Alan J.K. Millar from the Royal Botanic Gardens at Sydney, who, with D.W. Freshwater, initially reported *Gelidium isabelae* from Australia, and who has made significant contributions to the marine flora, including the Gelidiales in Australia.

Distribution: Australia.

Remarks: Millar & Freshwater^27^ identified this species as *Gelidium isabelae* on the basis of multiple shared morphological character states, including the presence of decussate lines on the flattened portions of blades in surface view. Our description is based on text and illustrations provided by Millar & Freshwater^25^,^26^,^27^.

***Gelidium coulteri*** Harv. 1853^34^.

Synonym: *Gelidium sinicola* N.L.Gardner (*Univ. Calif. Publ. Bot.* 13: 278, p. 47: Fig. 2. 1927^35^).

***Pterocladiella luxurians***(Collins) G.H.Boo & K.A.Mill. **comb. et stat. nov.**

Basionym: *Gelidium crinale* f. *luxurians* Collins (Collins, Holden & Setchell: Exsiccate No. 1138. 1903^36^; *Rhodora* 8: 111. 1906^37^).

Synonym: *Gelidium crinale* var. *luxurians* (Collins) N.L.Gardner (*Univ. Calif. Publ. Bot.* 13: 277, pl. 46: Fig. 1, pl. 47: Fig. 3. 1927^35^).

Remarks: *Gelidium crinale* f. *luxurians* is elevated to the species level.

***Pterocladiella media***(E.Y.Dawson) G.H.Boo & K.A.Mill. **comb. nov.**

Basionym: *Pterocladia media* E.Y.Dawson (*Bull. So. Calif. Acad. Sci.* 57: 68, pl. 21: Figs 3, 4, pl. 24: Fig. 11. 1958^38^).

***Pterocladiella musciformis***(W.R.Taylor) G.H.Boo & K.A.Mill. **comb. nov.**

Basionym: *Pterocladia musciformis* W.R.Taylor (*Allan Hancock Pac. Exped.* 12: 159. 1945^39^).

Synonym: *Gelidium musciforme* (W.R.Taylor) Santel. (*Pac. Sci.* 45: 4. 1991^40^).

## Discussion

Ours is the first study to analyze mitogenomes from archival type specimens in the Florideophyceae, which contains approximately 7,000 species^41^. The GC composition in *Gelidium* and *Pterocladiella* (as *Pterocladia*) (28.1% to 30.1%) is comparable to other Florideophyceae, as is the number of genes (43–44)^29^,^31^,^42^,^43^,^44^,^45^. The pattern of gene arrangement in the mitogenomes is identical among seven *Gelidium* species, and the genome sizes are similar (24,901 bp to 24,970 bp). This is also true of the five species in the genus *Pterocladiella* analyzed here. The Gelidialean mitogenomes have compact and conserved architectures of protein-coding, tRNA and rRNA genes, consistent with previous studies on florideophycean red algae^29^.

The present study provides the first report of Ka/Ks ratios for 23 protein-coding genes in the florideophycean red algae. The Ka/Ks values in the 12 *Gelidium* and *Pterocladiella* species analyzed here were all significantly lower than 1, indicating that purifying selection maintains their stability^46^. Interestingly, Ka/Ks ratios were highly variable among the 23 protein-coding genes and also variable among species for each gene (Fig. 4). *Sec*Y (0.3943), *sdh*D (0.3621), and *sdh*3 (0.3371) genes, with higher Ka/Ks values, have evolved more rapidly than other genes, while *atp*9 (0.0022), apparently under strong selection, has the lowest Ka/Ks. For unknown reasons, some genes (e.g., *sdh*3, *sdh*D, *sec*Y, *ymf*39) with high Ka/Ks ratios that are less than 800 bp and rich (>73%) in AT, are lost in some species of red algae, as is *nad*4L^29^, which has a lower Ka/Ks ratio.

Within the ATP synthase group, *atp*8 (0.2884) and *ymf*39 (0.2730) had higher ratios than *atp*6 (0.0583) and *atp*9 (0.0022), suggesting that there has been differential selection and that each gene evolved independently within the functional group. However, genes in the cytochrome c oxidase complex and apocytochrome b showed similar Ka/Ks ratios that were lower than other genes. The ratio was notably low in *cox*1 (0.0278), the barcoding gene widely used in protists including red algae^23^,^47^,^48^,^49^. Ka/Ks rations for *atp*6 (0.0583), *cob* (0.0494), *cox*2 (0.0509), *cox*3 (0.0610), *nad*1 (0.0439), and *nad*4L (0.0405) were similar to *cox*1 (0.0278). Six genes (*atp*6, *cob*, *cox*1, *cox*2, *cox*3 and *nad*1) may thus be suitable markers for detecting molecular evolution and phylogenetic structure in red algae. The Ka/Ks ratios in other genes are highly variable, indicating relaxed selective constraint and limiting their utility as markers in red algae.

The type mitogenomes generated in this study support previous genus-level phylogenetic studies of the Gelidiales^19^,^23^,^27^,^50^,^51^,^52^. Of the ten type specimens included here, five (*G. arborescens*, *G. galapagense*, *G. isabelae*, *G. sclerophyllum*, and *G. sinicola*) belong in the genus *Gelidium* (Fig. 4; Supplementary Figs S12–S14).

*Gelidium galapagense* and *G*. *isabelae* (type locality: Isla Isabela, Galápagos Islands) are both approximately 1 cm in height and were originally distinguished by their tetrasporangia-bearing branches, which in *G*. *galapagense* are on specialized branches that are irregularly palmately expanded from a constricted base, while tetrasporangial sori occur on ordinary branches in *G*. *isabelae*^39^. The *cox*1 and *rbc*L trees confirm that *G*. *galapagense* is distinct from *G*. *isabelae*. *Gelidium galapagense* formed a sister relationship with *G*. *coulteri*, whereas *G*. *isabelae* was nested in a clade with *G*. *arborescens*, *G*. *purpurascens*, and *G*. *robustum*. Our results suggest that *G. galapagense* and *G. isabelae* may be endemic to the Galápagos Islands.

We describe a new species, *G*. *millariana*, based on specimens previously identified as *G*. *isabelae* from Australia by Millar and Freshwater^27^. *Gelidium millariana* was consistently monophyletic and did not match the type of *G*. *isabelae* in our *cox*1 and *rbc*L sequence analyses. Rhizines are present in the subcortical layer in the type specimen of *G. isabelae*, but are abundant in both subcortical and medullary layers in *G. millariana*^27^,^39^. The morphological character states that were used to identify the Australian specimens as *G*. *isabelae* may be shared by many small, turfy *Gelidium* species and are not distinctive. *Gelidium isabelae* from South Africa, also misidentified by Millar & Freshwater^27^, will be discussed elsewhere.

Our data show that Korean *G. galapagense*^28^ is an evolutionarily distinct lineage, which we interpret as a new species. Although it is related to *G. millariana*, interspecific pairwise divergences between these two species were 3.0–3.1% in *cox*1, which is above intraspecific-level variation reported in previous studies^48^,^52^,^53^. Lanceolate tetrasporangial branches without sterile margins distinguish Korean *G*. *galapagense* from *G. millariana*, which has tetrasporangial branches with retuse tips and a sterile margin. A full taxonomic and nomenclatural revision of Korean *G. galapagense* will appear elsewhere.

*Gelidium sinicola* was described from Point Cavallo, San Francisco Bay, California^35^, but has not been reported since. Silva^54^, who searched for it without success at the type locality, doubted that it was distinct from *G. coulteri*, a species described from the Monterey Peninsula^34^ and common along the Pacific coast from British Columbia, Canada to Baja California, Mexico. Both *rbc*L and *cox*1 trees reveal that sequences from the type of *G*. *sinicola* are nearly identical to five *rbc*L and 31 *cox*1 sequences from *G*. *coulteri* specimens (Supplementary Figs S13, S14) distributed from Yaquina Bay, Oregon through the Monterey Peninsula to Baja California, Mexico. We confirm Silva’s suggestion and reduce *G*. *sinicola* to a later heterotypic synonym of *G*. *coulteri*.

In concatenated and individual trees, sequences from the type of *Gelidium arborescens* (type locality: Carmel Bay, Monterey County, California) are identical to those from a contemporary collection identified as *G*. *nudifrons* from southern California. The morphology of the two species is very similar^55^, but *G. nudifrons* has been collected only on the mainland and islands south of Point Conception (134 specimens in UC), while *G. arborescens* has been collected chiefly in Monterey and San Mateo counties (29 specimens in UC); three specimens collected in San Luis Obispo County were identified as *G. arborescens*, two of them from the drift. This range disjunction has been the most compelling rationale for retaining the species as separate^55^. Analysis of the mitogenomes from the type specimens of *G*. *nudifrons* is needed to confirm the relationships between *G*. *arborescens* and *G*. *nudifrons*.

Sequences from four contemporary specimens collected in Costa Rica closely matched (0.25% different) a partial *rbc*L sequence (398 bp) from the isotype of *Gelidium sclerophyllum* (type locality: Bahia San Francisco, Esmeraldas, Ecuador)^13^. The Costa Rican *G*. *sclerophyllum* sequences differed by 1.5% in *cox*1 and 0.9% in *rbc*L from the complete holotype specimen sequences generated in this study.

The genus *Pterocladiella* was established to accommodate species originally ascribed to *Pterocladia* that differed in the development of carpogonia, nutritive filaments, gonimoblast tissue, and carposporangia^56^. *Gelidium crinale* f. *luxurians*, *Pterocladia media*, *P. mexicana*, *P. musciformis*, and *P. robusta* grouped with *Pterocladiella* species in both the genome-derived analysis and concatenated tree. Thus, these species belong to the genus *Pterocladiella*. Our data do not support the merger of *Gelidium crinale* f. *luxurians* with *Pterocladia media* suggested by Stewart^24^ and accepted by Santelices^25^. *Pterocladia musciformis* was described from Golfo Dulce, Costa Rica by Taylor^39^. Santelices^40^ transferred this species to the genus *Gelidium* on the basis of cystocarp structure using samples from Golfo de la Union, El Salvador. A reexamination of cystocarp structure in sequence-verified specimens of *Pterocladiella musciformis* is warranted because the samples from El Salvador that Santelices^40^ studied may have been misidentified.

*Pterocladia mexicana* was originally described from Point Hughes, Cabo San Lanzaro, Baja California, Mexico by Taylor^39^; *Pterocladia robusta* was described from Punta Christopher, Isla Isabela, Galápagos Islands. Stewart^57^ merged both species with *P*. *capillacea*. We concur; our mitogenome and gene datasets strongly support the conspecificity of both *P. mexicana* and *P. robusta* with *Pterocladiella capillacea*, the generitype of *Pterocladiella*. Infraspecific diversity between *Pterocladiella capillacea* from the Galápagos Islands (as *P*. *robusta*) and Mexico (as *P*. *mexicana*) was 0.8% in COI-5P and 0.4% in *cox*1, and up to 1.1% in *nad*3 (Supplementary Table S4), all these values being well within population-level variation reported for red algae^30^,^48^,^53^.

Analysis of mitogenomes from type specimens greatly improved the resolution of evolutionary relationships at the generic and specific levels in the Gelidiaceae and Pterocladiaceae. As a result, we are able to describe a new species, *Gelidium millariana* that has been misidentified as *G*. *isabelae* from Australia, to transfer three species of *Gelidium* and *Pterocladia* to the genus *Pterocladiella*, and to merge *Gelidium sinicola* with the earlier name, *G*. *coulteri*. The species studied here were originally described and delineated on the basis of morphological characters, but our molecular data based on type material revealed that the majority of identifications of these species were incorrect. Conflicts between morphological- and molecular-based methods result from the lack of visible diagnostic characters in these species with simple architecture and anatomy.

## Conclusions

Herbaria, treasure houses of DNA, are major resources for species discovery^58^. Type specimens of seaweeds have great value for current and future systematic and nomenclatural studies. This is also true for fungi, bryophytes and other morphologically simple organisms. Sequencing DNA from type specimens may not be possible for all red algae (more than 7,000 species) or other taxa. When type specimens cannot be sequenced (e.g., because they are poorly preserved or lost), topotype material can be used. We have shown that complete mitogenome sequences from type specimens are invaluable for constructing phylogenies and for applying the correct names to clades and can potentially serve as standard surrogates for type specimens that will continue to degrade with time. We foresee that type mitogenomes will provide vital information for deciphering the phylogeography of cosmopolitan species for which species boundaries are unclear. Most type specimens are the oldest representatives of the species; as such, type mitogenomes, when compared to contemporary samples, will provide insight into the results of selection, bottlenecks, anthropogenic dispersal, and expansion or reduction of species ranges due to climate change.

To expand the utility of analyzing mitogenomes from archival type specimens, we propose that i) type mitogenomes will provide important independent, objective characters, especially for morphologically simple organisms, ii) curators share fragments of type specimens, if available, for NGS sequencing, because these mitogenomes increase the value of the specimens, and iii) mitogenomes from type specimens should be maintained as an independent, international “type mitochondrial genomes” domain in GenBank for convenient public communication.

## Methods

### Type fragments and extraction of DNA

Ten species in the Gelidiaceae were selected for sequencing the mitogenome: *Gelidium arborescens*, *G*. *crinale* f. *luxurians*, *G*. *galapagense*, *G*. *isabelae*, *G*. *sclerophyllum*, *G*. *sinicola*, *Pterocladia media*, *P*. *mexicana*, *P*. *musciformis*, and *P*. *robusta*. An apical fragment approximately 5 mm in size was isolated from each of ten type specimens housed in the UC (Supplementary Table S1).

DNA was extracted from type material with strict adherence to the precautionary guidelines outlined by Hughey & Gabrielson^12^ using the DNeasy Blood & Tissue Kit (Qiagen, Valencia, California, USA). Tissue was pre-incubated for 30 min at 55 °C in 2 ml tube in a solution of 237 μl of Buffer ATL and 27 μl of Proteinase K. Partially digested samples were ground with the wooden end of sterile swab and further incubated for 30 min to 2 h. Samples were centrifuged for 3 min at 13,000 rpm and the supernatant was transferred to a new tube. 200 μl of 100% ethanol and 200 μl Buffer AL were added. The mixed sample was transferred to the DNeasy spin column and centrifuged for 2 min at 8,000 rpm. The spin column was removed and placed in a new 2 ml collection tube and washed with 500 μl of Buffer AW1 by centrifugation for 1 min at 8,000 rpm. The column was then placed in a new 2 ml collection tube and washed with 500 μl of Buffer AW2 by centrifugation for 3 min at 13,000 rpm. The DNA was eluted with 60 μl of Buffer AE for 5 min at room temperature and centrifuged for 1 min at 8,000 rpm. DNA quality and quantity were analyzed by the University of Washington High-Throughput Genomics Unit (UW-HTGU, Seattle, USA) on an Agilent 2100 Bioanalyzer^TM^ following the manufacturer’s instructions.

### High-throughput sequencing and assembly

The genome library was constructed using a modified TruSeq protocol developed by UW-HTGU^2^. The 36 bp single end sequencing analysis was performed by UW-HTGU using the manufacturer’s protocol via the cBot and HiSeq 2000. Filtered reads were base called using Illumina’s standard pipeline, then assembled using Velvet v.1.2.10 59 and CLC Genomic Workbench (2014 CLC bio, a QIAGEN Company). For the Velvet assemblies, data from the first run (kmers = 31, 29, 27, 25, with autosettings) were used to optimize the expected cutoff and coverage cutoff for the second run. The resulting contigs from the assemblies were searched at the National Center for Biotechnology Information (NCBI) using Megablast; aligned contigs were ordered according to previously published sequences of *G*. *elegans* and *G. vagum*^29^,^31^. Joined contigs were validated by PCR amplification and sequencing, or by mapping to reference sequences using Geneious R7 60.

### Genome annotation and comparative analysis

The open reading frames (ORFs) were annotated using NCBI ORF-finder and alignments obtained via BLASTX and BLASTN searches at NCBI. The tRNAs were identified using the tRNAscan-SE 1.21 web server^61^ and the rRNAs using the RNAmmer 1.2 server^62^. The physical map of the mitogenome was prepared for visualization using OrganellarGenomeDRAW (OGDraw)^63^, and compared using Blast Ring Image Generator (BRIG)^64^. Locally collinear blocks (LCBs) alignments were generated using ProgressiveMauve^65^ with a seed of 21 for the mitochondrial alignments and the ‘Use seed families’ option selected. Twenty-three protein-coding genes were aligned separately using the ClustalW translational alignment function in Geneious^60^ including all available mitogenomes of the Gelidiales and *Cyanidioschyzon merolae*, *Chondrus crispus*, *Hildenbrandia rubra* and *Pyropia perforata* as outgroups^2^,^29^,^42^,^66^. The protein alignments were concatenated and poorly aligned regions were removed using the Gblocks server^67^, with the less stringent selection options, reducing the alignment from 6,182 to 5,589 position. ProtTest 3.4.2 68 was used to select the best-fitting models of molecular evolution based on Akaike Information Criterion (AIC). The phylogeny was inferred by the ML method using RAxML v8.0.X^69^ with the rapid bootstrap analysis, and searching for the best-scoring ML tree in one single program run with 1,000 bootstrap replicates under the CpREV + G + I + F model. The Bayesian inference (BI) was performed with MrBayes v.3.2.1 70 using the Metropolis-coupled Markov Chain Monte Carlo (MC^3^) based on the CpREV + G + I + F model. For each matrix, two million generations of two independent runs were performed with four chains and sampling trees every 200 generations. The burn-in period was identified graphically by tracking the likelihoods at each generation to determine whether they reached a plateau. Ten percent of saved trees were removed, and the remaining trees were used to calculate the Bayesian posterior probabilities (BPP). Pairwise divergences within *Gelidium*, *Pterocladiella*, and two specimens of *P*. *capillacea* were measured using MEGA6^71^. To test the selection pressure of mitochondrial protein coding genes, ratios of nonsynonymous (Ka) versus synonymous substitutions (Ks) were measured using DnaSP 5.10^72^.

### Analysis of *cox*1 and *rbc*L from contemporary specimens

Genomic DNA was extracted from ~5 mg of dried tissue (Supplementary Table S5) ground in liquid nitrogen using the NucleoSpin Plant II Kit (Macherey-Nagel, Düren, Germany) according to the manufacturer’s protocol. Primer pairs for the amplification and sequencing were COXI43F-COXI1549R for *cox*1^73^ and F7-R753 and F645-RrbcS for *rbc*L^18^,^74^,^75^. PCR reactions were carried out in a volume of 10 μl, containing 5 μl of 2X Quick Taq HS DyeMix (Toyobo, Osaka, Japan), 0.2 μl primer (each), 1 μl of genomic DNA, and sterile deionized water. The cycle parameters were set as follows: a preliminary denaturation step of 94 °C for 2 min, followed by 40 cycles of 30 sec at 94 °C, 30 sec at 50 °C, and 1 min at 68 °C. PCR products were purified by enzymatic treatment with Exonuclease (Exo) and Antarctic Phosphatase (AP) (Exo-AP PCR Clean-Up Mix, Doctor Protein, Korea). Sequencing of the forward and reverse strands of the purified PCR products was performed by Genotech (Daejeon, Korea). The sequences were edited using Chromas v.1.45 and rechecked manually. Sequences were aligned with MEGA6 71.

Phylogenies of individual and combined datasets were inferred using ML and BI. PartitionFinder v1.1.0 76 was used to select the best-fitting partitioning schemes and models of molecular evolution using the greedy algorithm with unlinked branch lengths. The best-fitting substitution model as evaluated by PartitionFinder for the individual and combined (*cox*1 + *rbc*L) datasets, identified the GTR + G + I model. The ML analyses were performed using the Pthreads version of RAxML v8.0.X^69^ with the rapid bootstrap analysis, and searching for the best-scoring ML tree in one single program run with 1,000 bootstrap replicates under the GTR + G + I substitution model.

The BI was performed for individual and combined datasets with MrBayes v.3.2.1^70^ using the MC^3^ based on the best-fitting partitioning scheme and substitution models as evaluated with PartitionFinder. For each matrix, two million generations of two independent runs were performed with four chains and sampling trees every 100 generations. The burn-in period was identified graphically by tracking the likelihoods at each generation to determine whether they reached a plateau. Twenty-five percent of saved trees were removed, and the remaining trees used to calculate the BPP.

## Additional Information

**How to cite this article**: Boo, G. H. *et al*. Mitogenomes from type specimens, a genotyping tool for morphologically simple species: ten genomes of agar-producing red algae. *Sci. Rep.* **6**, 35337; doi: 10.1038/srep35337 (2016).

## Supplementary Material

Supplementary Information
