# Supplementary material for: Mitogenomes from type specimens, a genotyping tool for morphologically simple species: ten genomes of agar-producing red algae
Source: Sci Rep. 2016 Oct 14;6:35337. doi: 10.1038/srep35337 (PMC5064358; doi:10.1038/srep35337)
Supplement: Supplementary Information [file srep35337-s1.pdf]

Supplementary Information

Mitogenomes from type specimens, a genotyping tool for morphologically simple species: ten genomes of agar-producing red algae

Ga Hun Boo, Jeffery R. Hughey, Kathy Ann Miller, Sung Min Boo

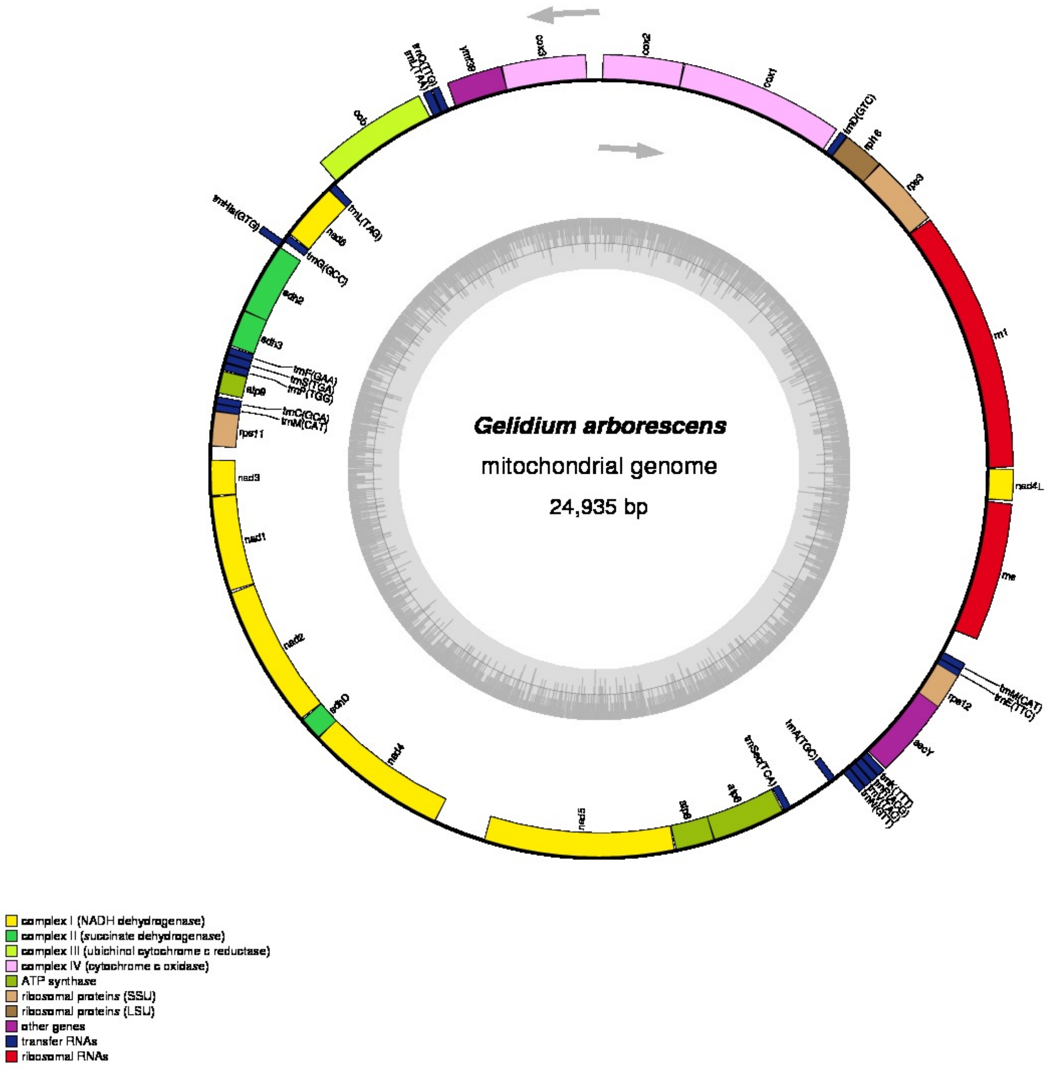

Figure S1. The mitogenome map of *Gelidium arborescens*. Color indicates difference gene classification.

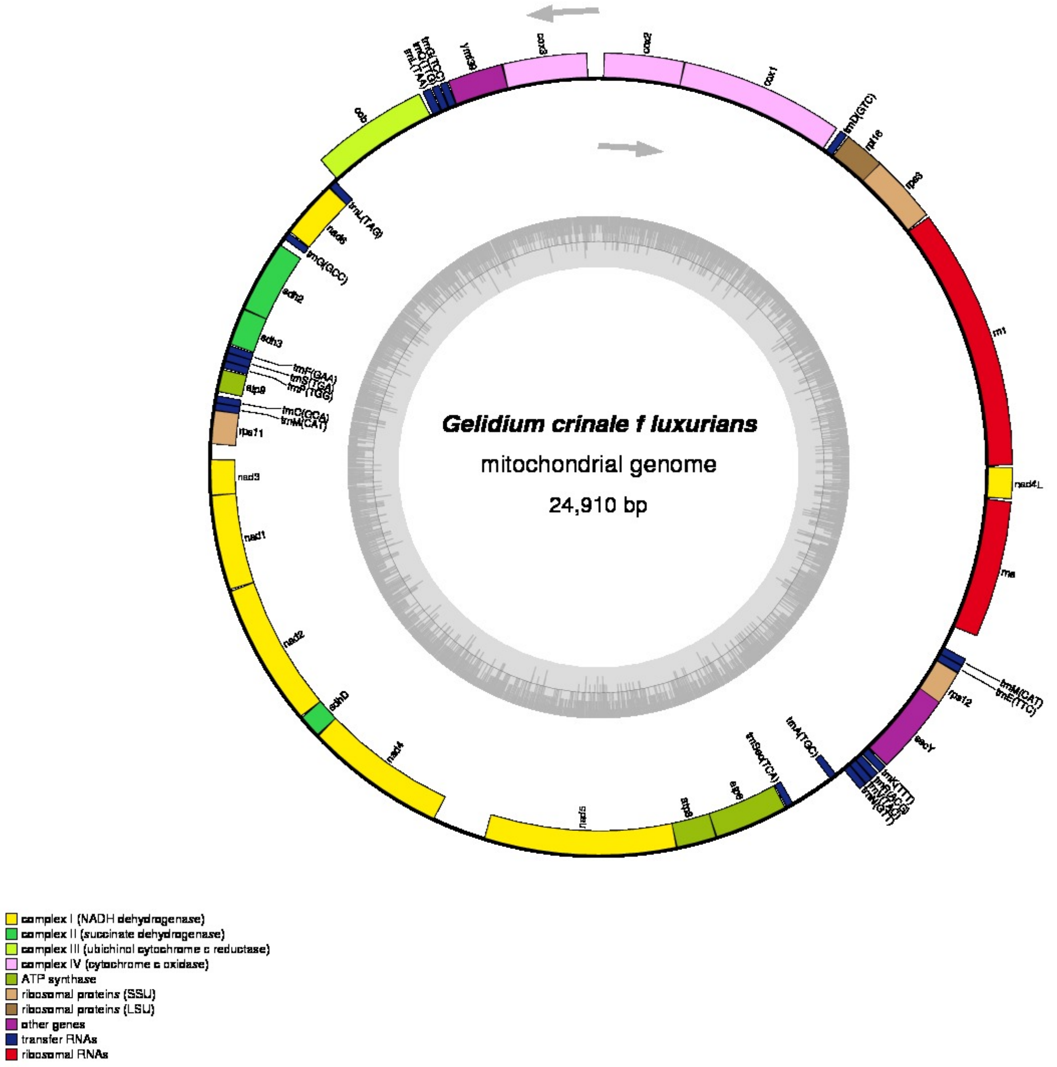

Figure S2. The mitogenome map of *Gelidium crinale* f. *luxurians*. Color indicates gene classification.

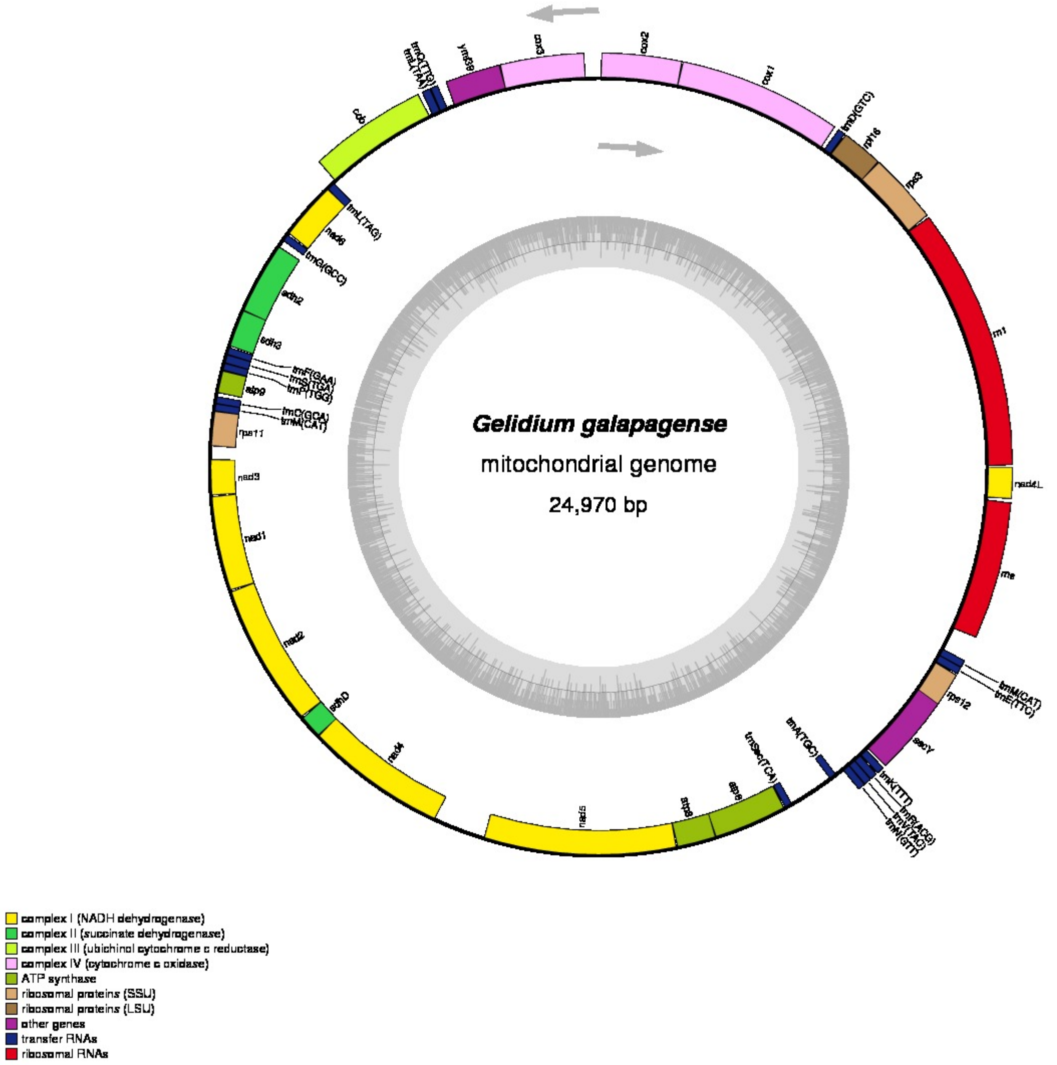

Figure S3. The mitogenome map of *Gelidium galapagense*. Color indicates gene classification.

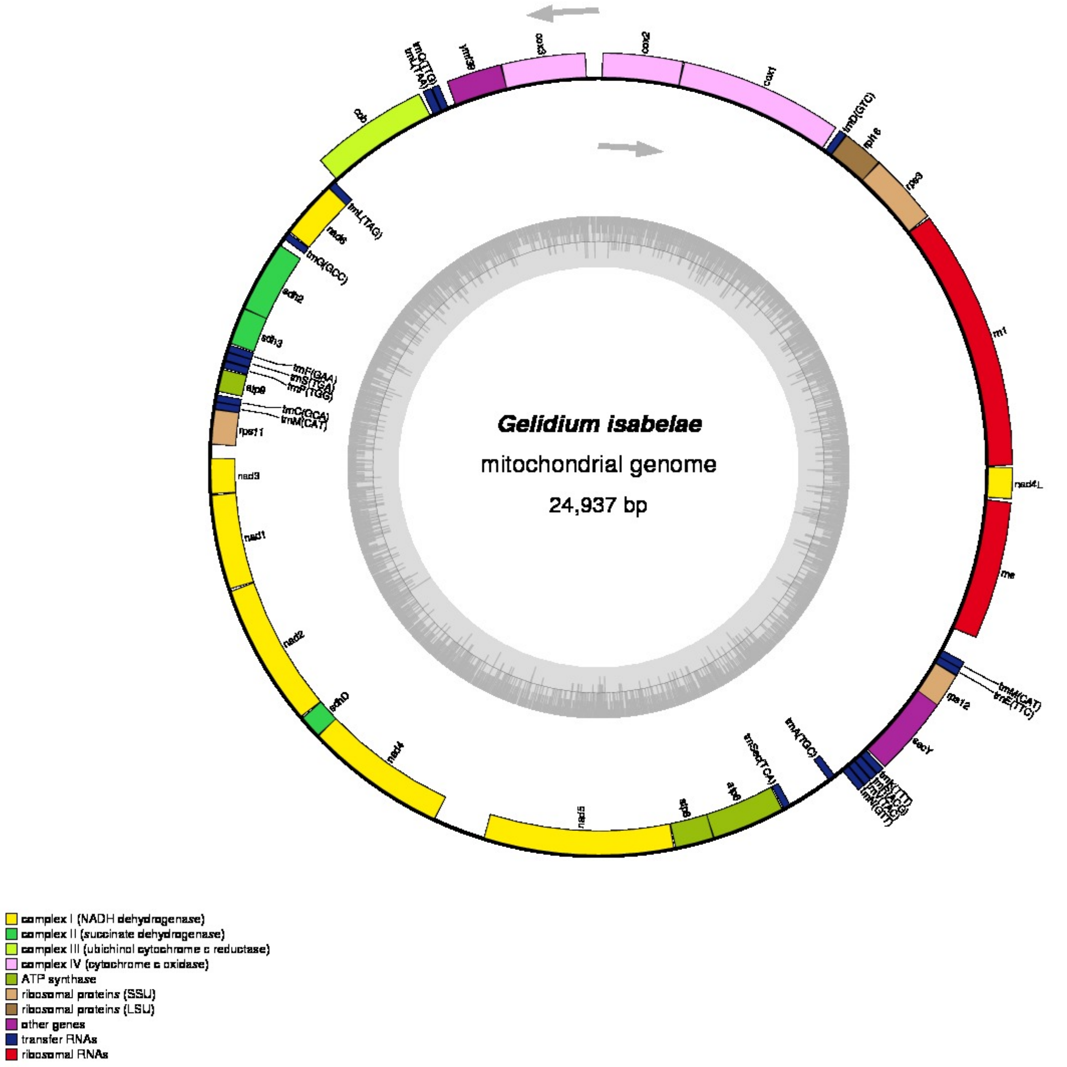

Figure S4. The mitogenome map of *Gelidium isabelae*. Color indicates gene classification.

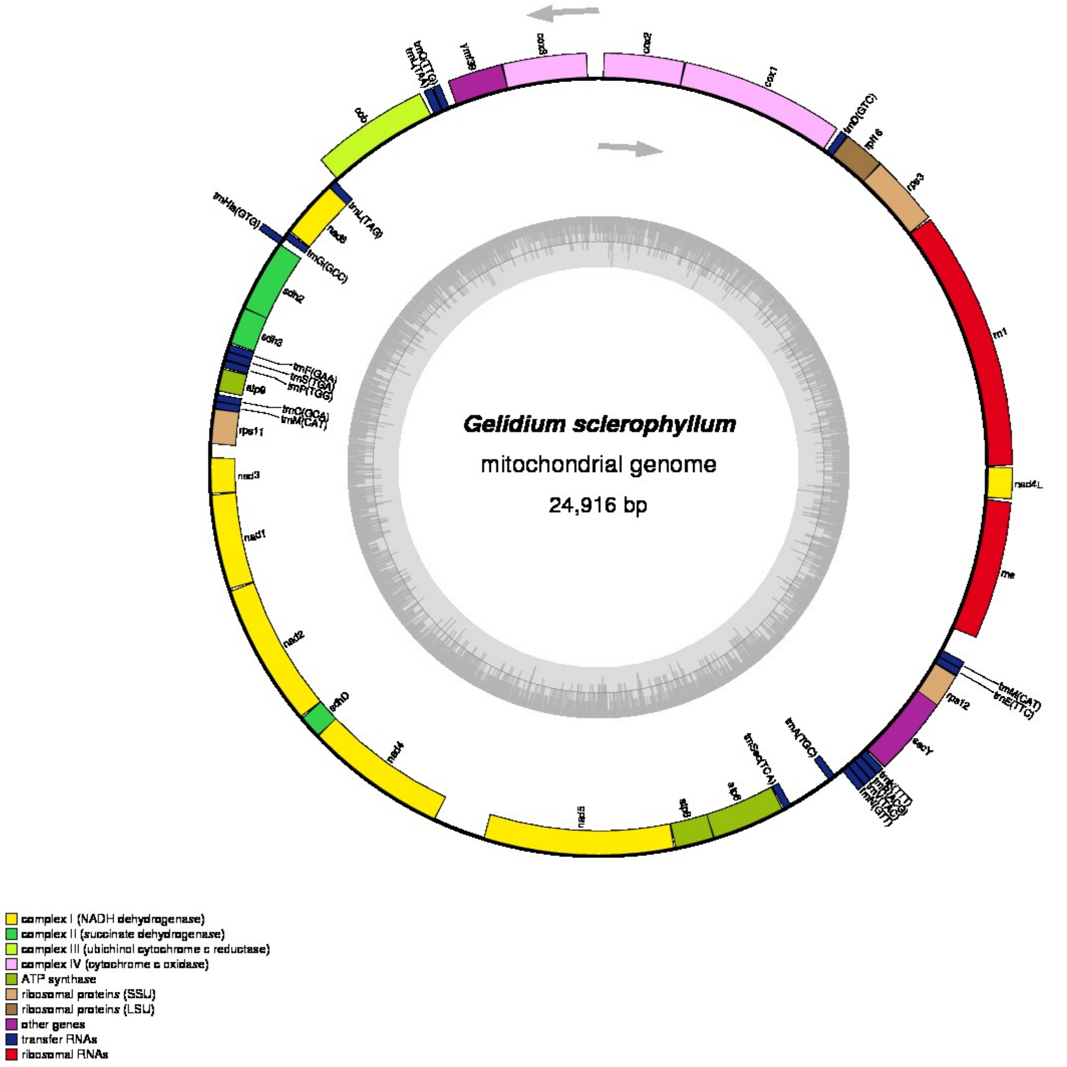

Figure S5. The mitogenome map of *Gelidium sclerophyllum*. Color indicates gene classification.

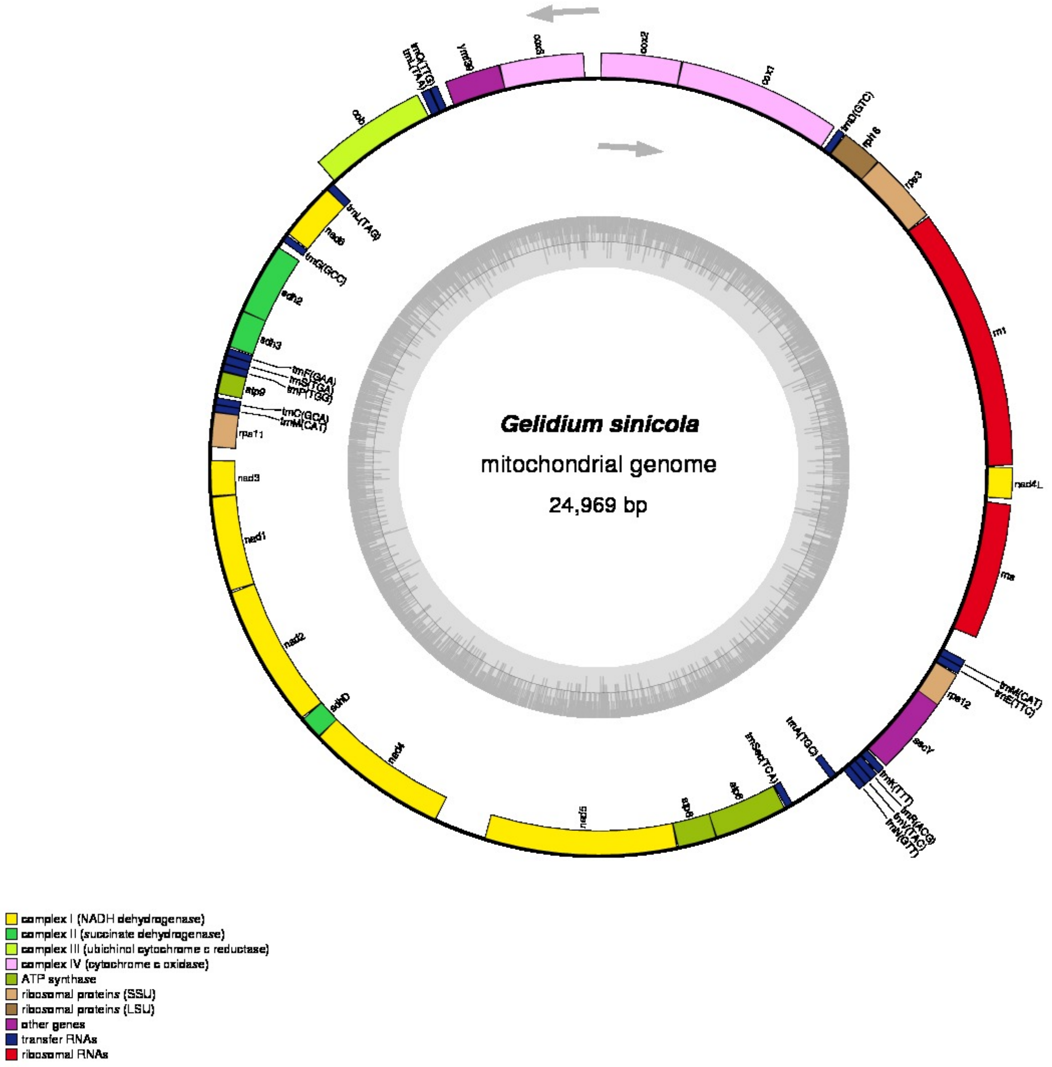

Figure S6. The mitogenome map of *Gelidium sinicola*. Color indicates gene classification.

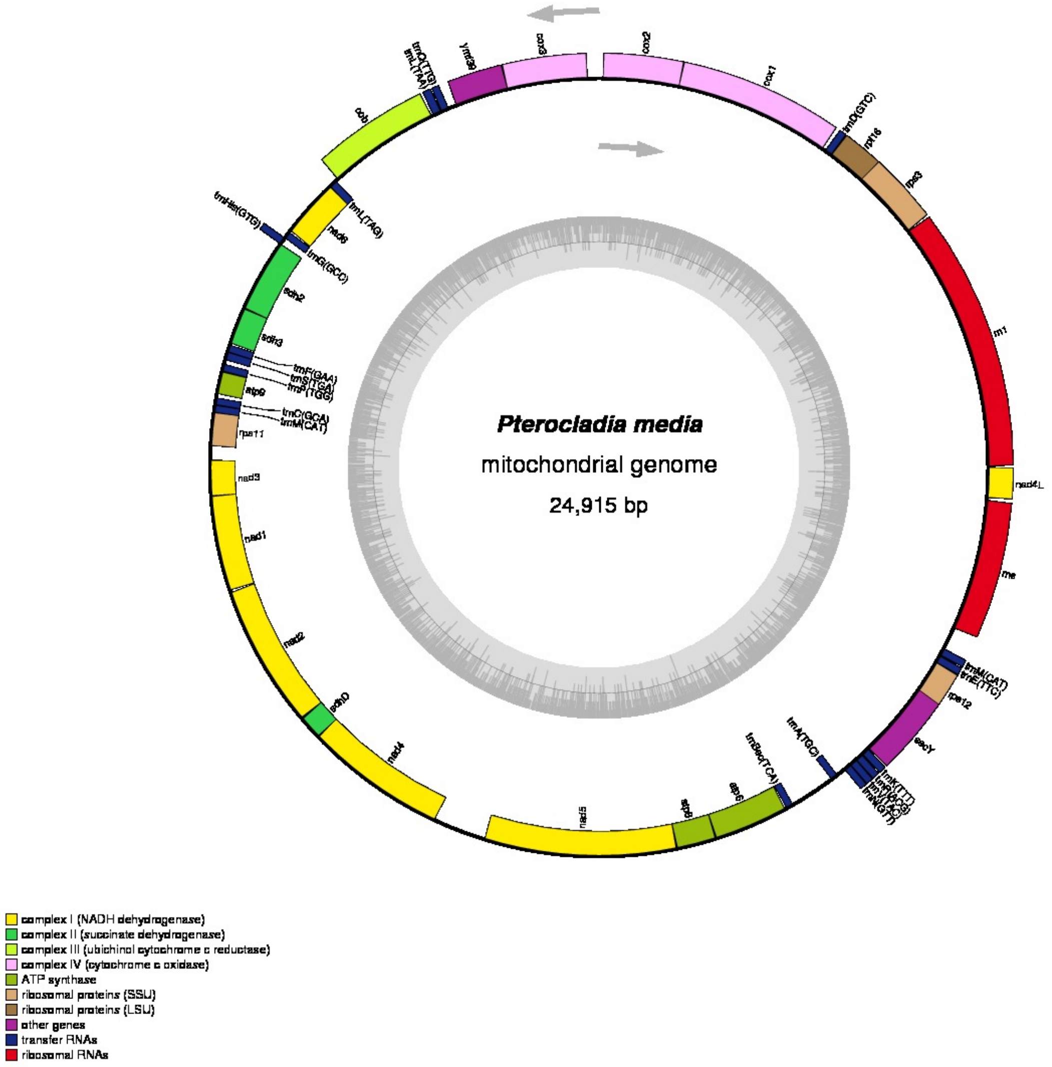

Figure S7. The mitogenome map of *Pterocladia media*. Color indicates gene classification.

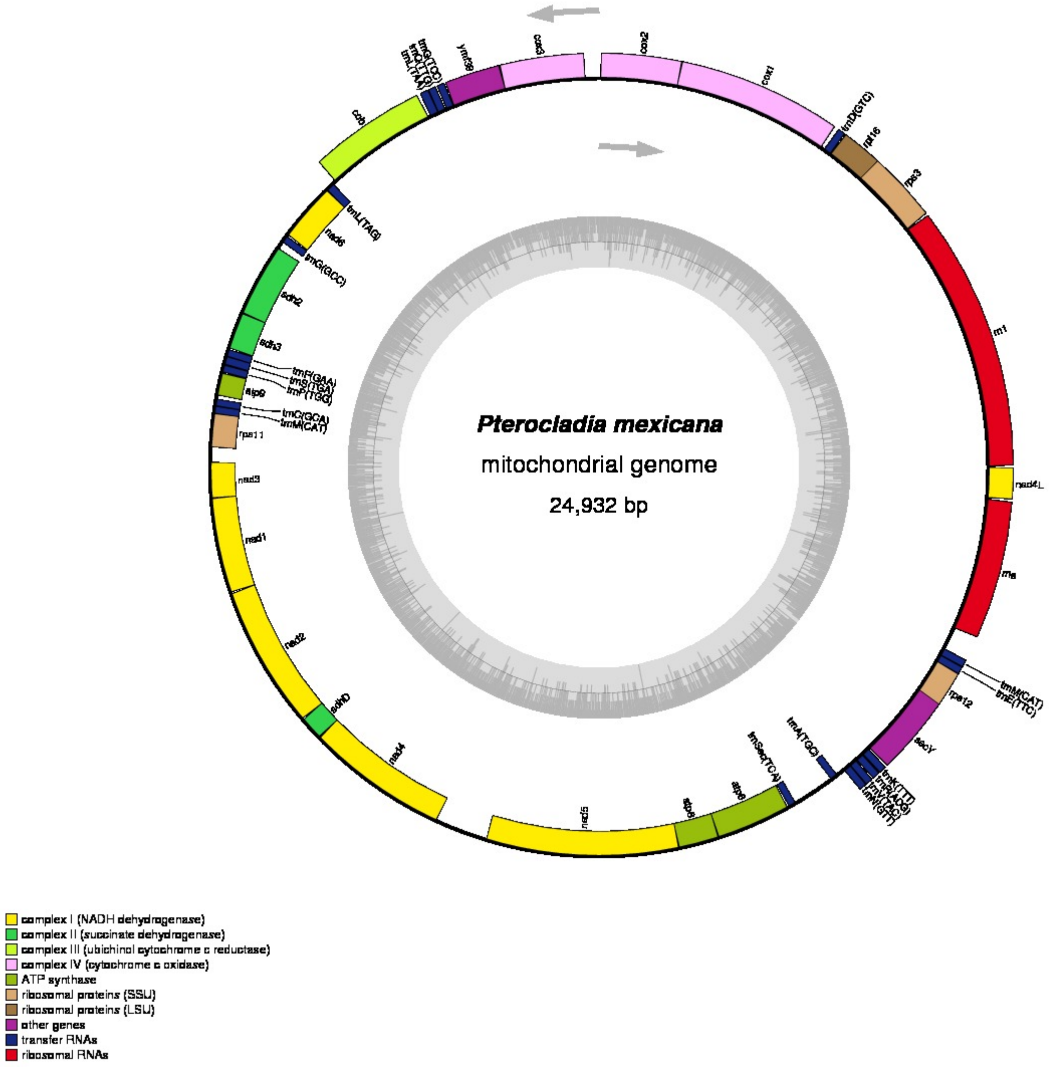

Figure S8. The mitogenome map of *Pterocladia mexicana*. Color indicates gene classification.

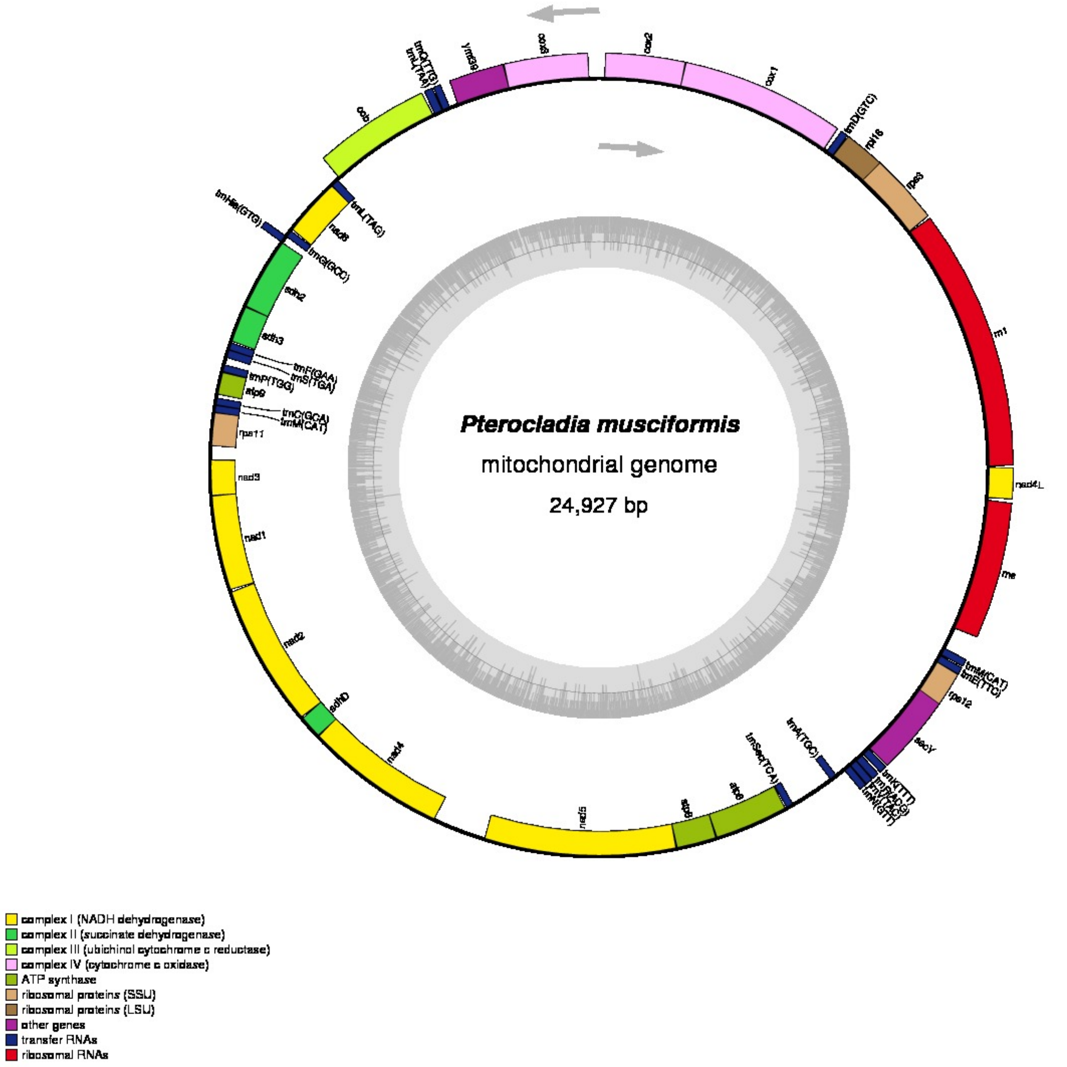

Figure S9. The mitogenome map of *Pterocladia musciformis*. Color indicates gene classification.

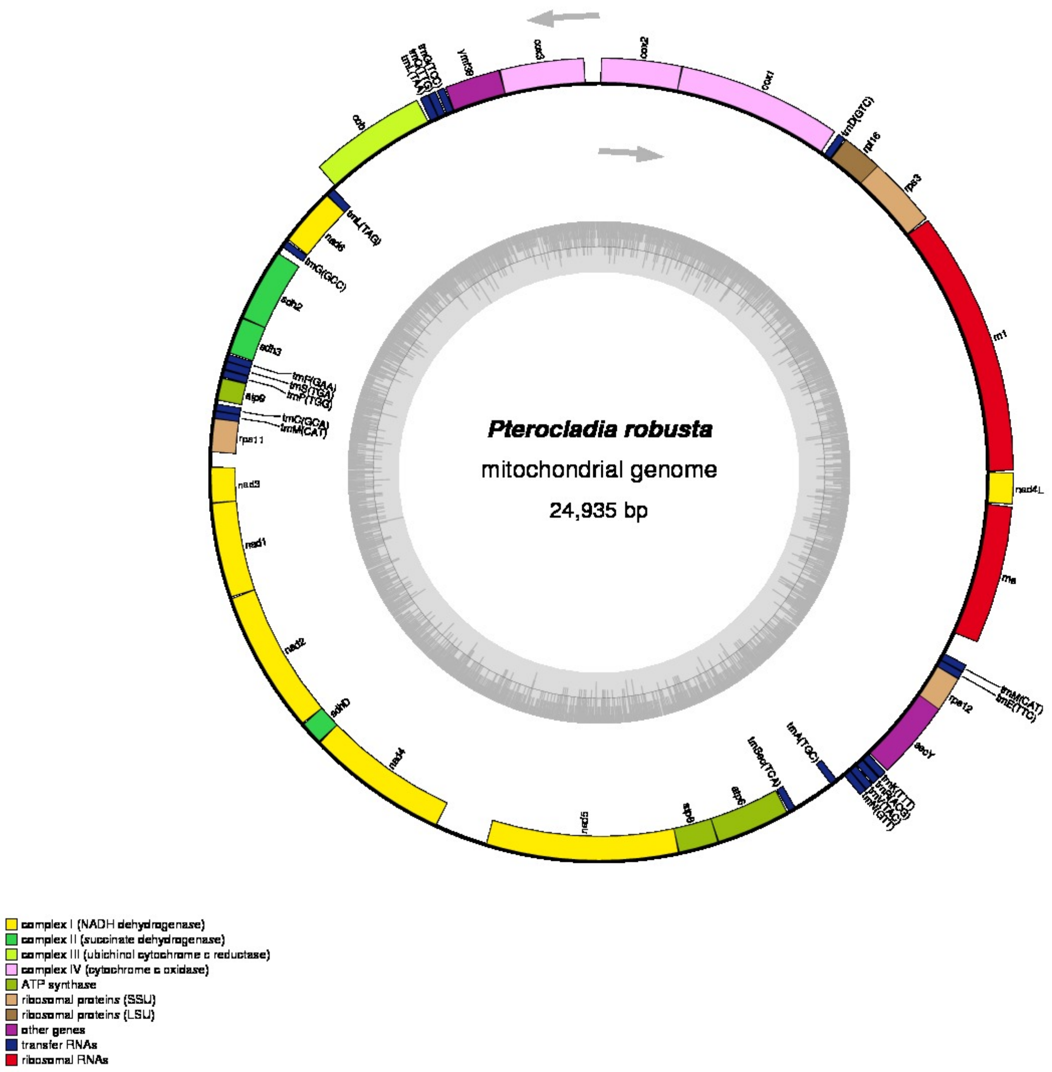

Figure S10. The mitogenome map of *Pterocladia robusta*. Color indicates gene classification.

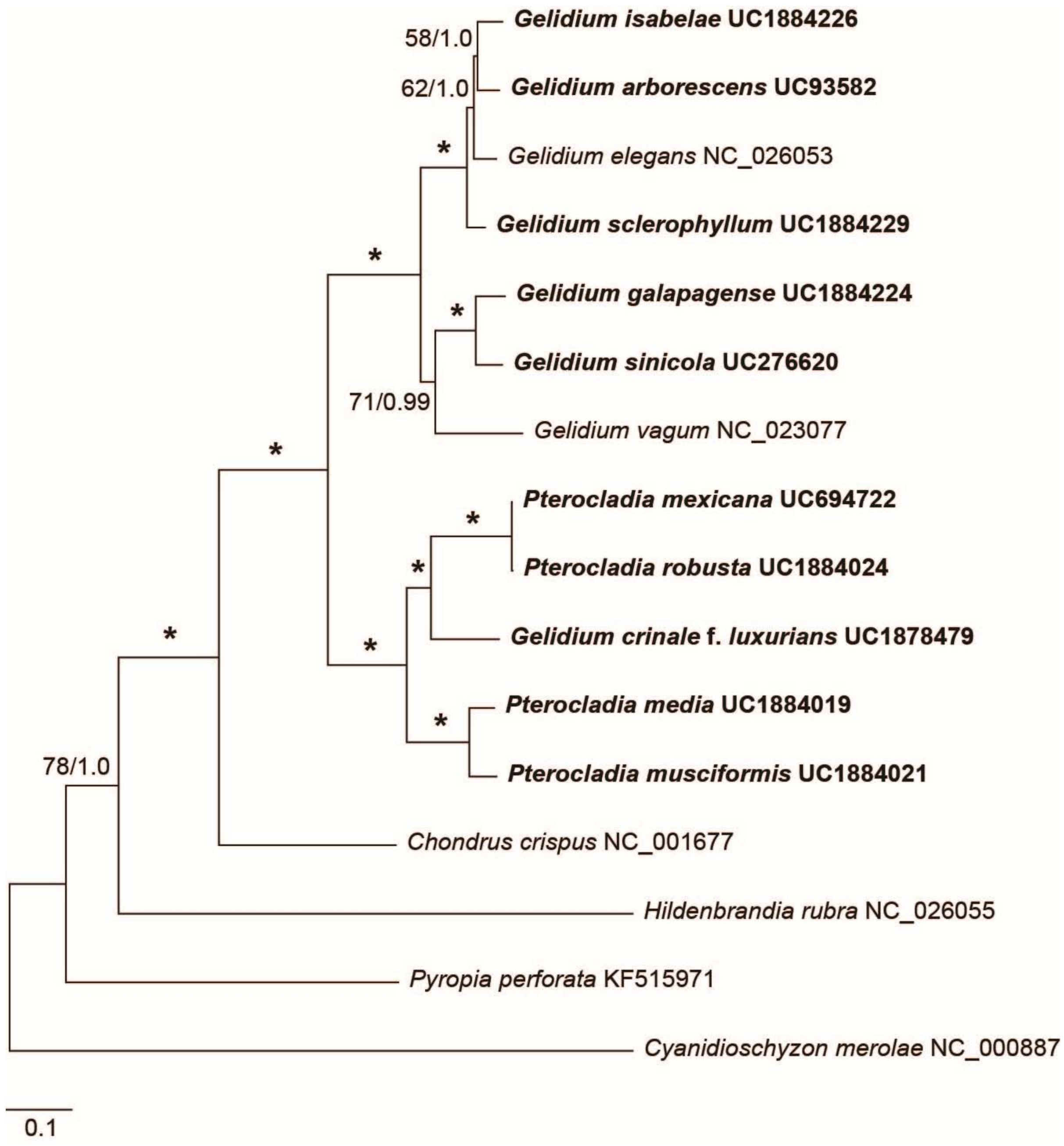

Figure S11. Maximum likelihood tree of mitogenome sequences from 12 species in the Gelidiales. Tree constructed based on 5,589 amino acids from 23 protein-coding genes using the CpREV + G + I + F model. Statistically supported bootstrap values (≥50%) and Bayesian posterior probabilities (≥0.90) are shown. Asterisks indicate full support in both analyses.

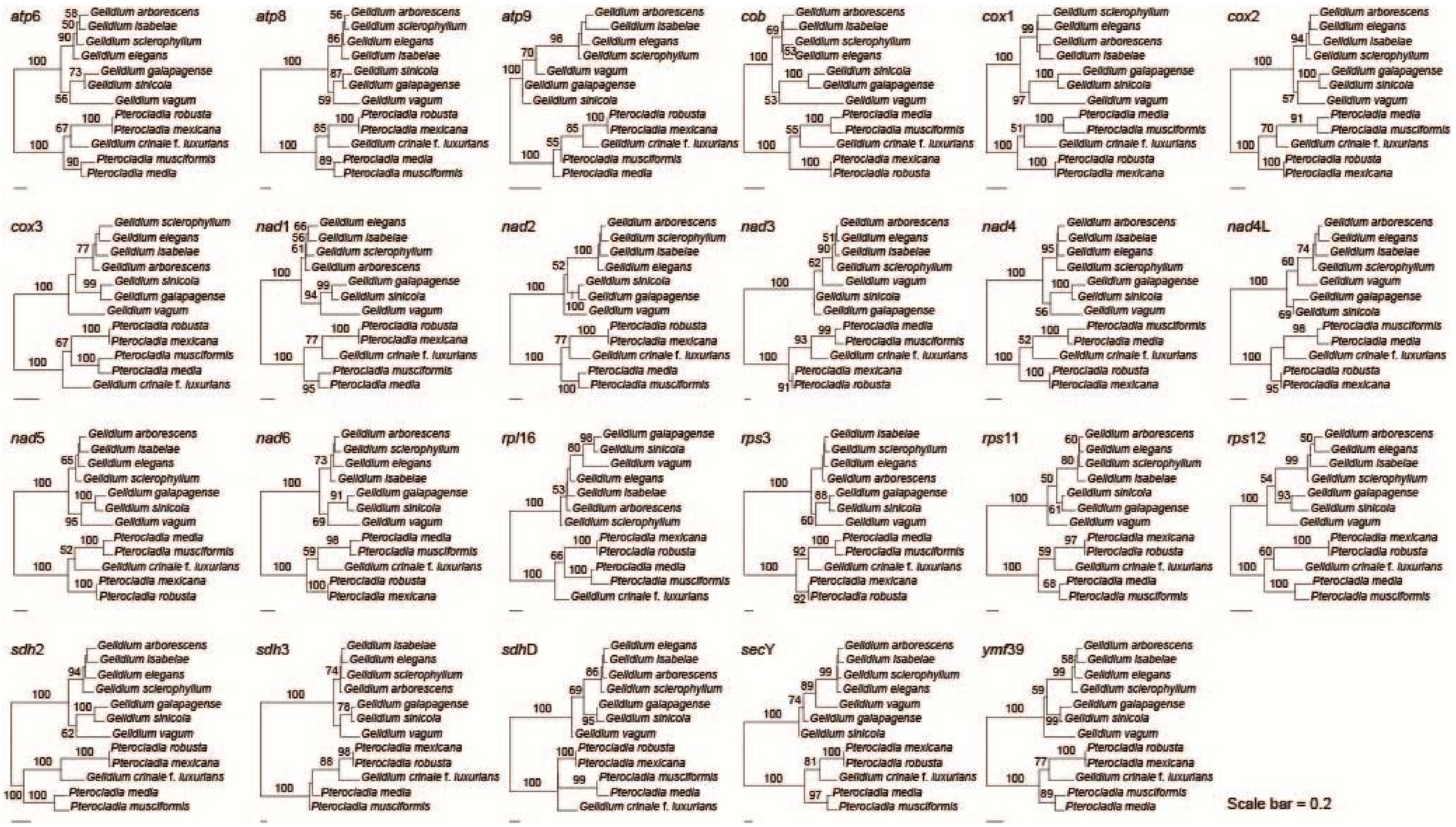

Figure S12. Maximum likelihood trees of 23 individual gene sequences from 12 Gelidiales species using the GTR + G + I model. Statistically supported bootstrap values (≥50%) are shown. Scale bar indicate 0.2 nucleotide substitutions per site.

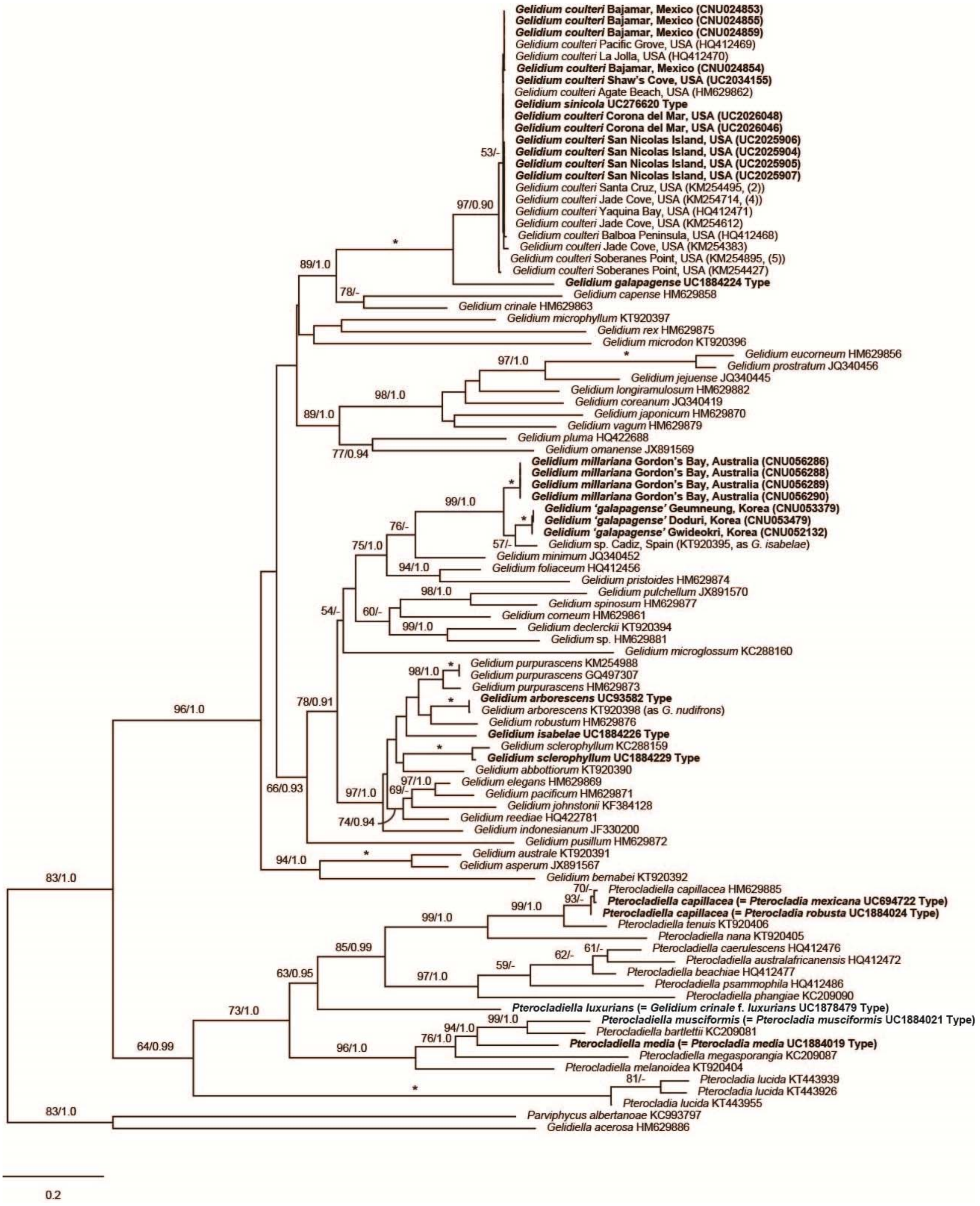

Figure S13. Maximum likelihood tree of *cox*1 sequences from *Gelidium*, *Pterocladia* and *Pterocladiella* using the GTR + G + I model. Statistically supported bootstrap values (≥50%) and Bayesian posterior probabilities (≥0.90) are shown. Asterisks indicate full support in both analyses. The numbers in parentheses after sequence designations represent the number of specimens with that identical sequence.

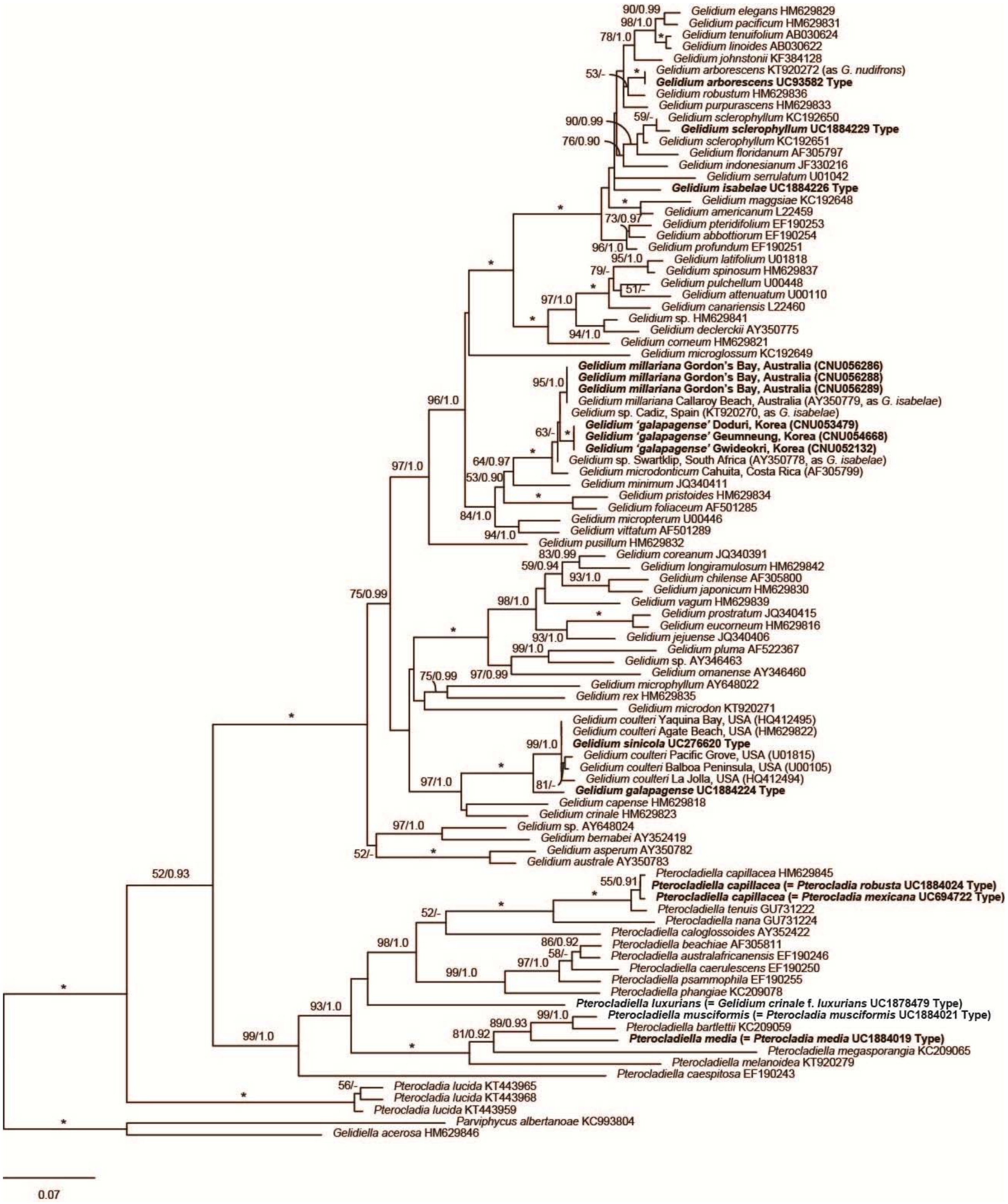

Figure S14. Maximum likelihood tree of *rbc*L sequences from *Gelidium*, *Pterocladia* and *Pterocladiella* using the GTR + G + I model. Statistically supported bootstrap values (≥50%) and Bayesian posterior probabilities (≥0.90) are shown. Asterisks indicate full support in both analyses.

Table S1. Taxon name, synonym, type information, publication of species name, habit, and determination of species in the present study.

Taxon	Synonym	Type information	Published in	Habit	Determination in the present study
Gelidium arborescens N.L.Gardner 1927	-	UC93582 (*W.A. Setchell*); 17 May 1897; Growing in subtidal belt, Carmel Bay, Monterey County, California, USA	Univ. Calif. Publ. Bot. 13: 276, pl. 42.	Plants up to 25 cm, cartilaginous and rigid; axes cylindrical, with few short branches; branches of all orders arising normally from the flattened surfaces; tetrasporiferous ramuli short, unbranched	*Gelidium arborescens* N.L.Gardner
Gelidium crinale f. *luxurians* Collins 1906	*Pterocladia media* by Stewart (1974)	UC1878479 (*E. Snyder*); 8 Mar. 1899; On stones at low tide mark, Pacific Beach, San Diego County, California, USA	Phycol. Bor.-Amer. No. 1138, 1903; Rhodora 8: 111. 1906.	Plants up to 6.5 cm, fragile; axes chiefly distichous; branches opposite or alternate; terminal ramuli almost cylindrical; medullary cells thick-walled	*Pterocladiella luxurians* (Collins) G.H.Boo & K.A.Mill. comb. et stat. nov.
Gelidium galapagense W.R.Taylor 1945	-	UC1884224 (*W.R. Taylor 34-130*); 13 Jan. 1934; Banks Bay, Isla Isabela, Galápagos Islands, Ecuador	Allan Hancock Pac. Exp. 12: 155, pl. 5, figs 2-6.	Plants up to 1 cm, highly irregular tetrasporangial branchlets, which are fanlike to irregularly digitate	*Gelidium galapagense* W.R.Taylor. Korean *G. galapagense* was misidentification; its identity will be published elsewhere.
Gelidium isabelae W.R.Taylor 1945	-	UC1884226 (*W.R. Taylor 34-121*); 12 Jan. 1934; Pt. Albemarle, Isla	Allan Hancock Pac. Exp. 12: 154-155, pl. 5, figs 8-12.	Plants up to 1 cm, fleshy, firm rather than membranous texture and simple form	*Gelidium isabelae* W.R.Taylor. Australian *G. isabelae* is *Gelidium millariana*

		Isabela, Galápagos Islands, Ecuador			G.H.Boo, Hughey, K.A.Mill. & S.M.Boo sp. nov.
Gelidium sclerophyllum W.R.Taylor 1945	-	UC1884229 (*W.R. Taylor 34-489*); 11 Feb. 1934; near northern limit of Bahia San Francisco, Esmeraldas, Ecuador	Allan Hancock Pac. Exp. 12: 156, pl.5, fig. 13, pl. 33, fig. 2.	Plants up to 3 cm, 1-2 pinnate erect branches arising from base; tetrasporangial branchlets with a thickened margin, notably large thick-walled cells evident in sections	*Gelidium sclerophyllum* W.R.Taylor
Gelidium sinicola N.L.Gardner 1927	-	UC276620 (*Gardner 2615*); Nov. 1913; Point Cavallo, San Francisco Bay, California, USA	Univ. Calif. Publ. Bot. 13: 278, pl. 47, fig. 2.	Plants up to 6 cm, axes cylindrical to subcylindrical, abundantly branched; branches considerably contorted; medullary cells large and thick-walled; rhizines mostly in medullary layer	Merged with *G. coulteri* Harv.
Pterocladia media E.Y.Dawson 1958	*Gelidium crinale* f. *luxurians* by Stewart (1974) & Santelices (1999)	UC1884019 (*Dawson 15609*); 1 Dec. 1956; intertidal above the reef flat, opposite the north end of Neptune Place, La Jolla, California, USA	Bull. South Calif. Acad. Sci. 57: 68, pl. 21, figs 3, 4; pl. 24, fig. 11	Plants up to 3 cm, erect branches arising from stoloniferous, creeping branch with short peg-like holdfasts; erect axes with closely spaced pinnae, rhizines sparsely scattered in medullary layer; reproductive structures unknown	*Pterocladiella media* (E.Y.Dawson) G.H.Boo & K.A.Mill. comb. nov.

Pterocladia mexicana W.R.Taylor 1945	*Pterocladiella capillacea* by Santelices (1999)	UC694722 (*W.R. Taylor 34-601*); 7 Mar. 1934; Point Hughes, Cabo San Lazaro, Baja California, Mexico	Allan Hancock Pac. Exp. 12: 159-160, p. 35.	Plants up to 1 cm, very bushy from the base, 3-4 times regularly branched, becoming triangular, complanate; rhizines chiefly in medulla; tetrasporangia in decussate rows	Confirmed previous merger with *P. capillacea*.
Pterocladia musciformis W.R.Taylor 1945	*Gelidium musciforme* by Santelices (1991)	UC1884021 (*W.R. Taylor 39-106*); 26 Mar. 1939; Golfo Dulce, Costa Rica	Allan Hancock Pac. Exp. 12: 159.	Plants up to 1 cm, forming moss-like turfs	*Pterocladiella musciformis* (W.R.Taylor) G.H.Boo & K.A.Mill. comb. nov.
Pterocladia robusta W.R.Taylor 1945	*Pterocladiella capillacea* by Stewart (1968)	UC1884024 (*W.R. Taylor 34-198*);16 Jan. 1934; Pt. Christopher, Isla Isabela, Galápagos Islands, Ecuador	Allan Hancock Pac. Exp. 12: 160, pl. 36.	Plants up to 15 cm; main axes to 2.5 mm wide, lower axis naked, above 2-4 times regularly branched, with ligulate to spatulate branchlets	Confirmed previous merger with *P. capillacea*.

Table S2. Characteristics of complete mitogenomes in the Gelidiales.

Species	Size (bp)	G+C (%)	Protein-coding genes	tRNAs	rRNAs	Total genes	Reference
Gelidium arborescens	24,935	29.1	23	19	2	44	This study
Gelidium crinale f. *luxurians*	24,910	28.7	23	19	2	44	This study
Gelidium elegans	24,922	29.5	23	19	2	44	Yang et al. (2015)
Gelidium galapagense	24,970	30.0	23	18	2	43	This study
Gelidium isabelae	24,937	29.2	23	18	2	43	This study
Gelidium sclerophyllum	24,916	29.6	23	19	2	44	This study
Gelidium sinicola	24,969	30.2	23	18	2	43	This study
Gelidium vagum	24,901	30.5	23	18	2	43	Yang et al. (2014)
Pterocladia media	24,915	28.2	23	19	2	44	This study
Pterocladia mexicana	24,932	29.9	23	19	2	44	This study
Pterocladia musciformis	24,927	28.1	23	19	2	44	This study
Pterocladia robusta	24,935	29.8	23	19	2	44	This study

Table S3. A comparison of tRNAs in Gelidiales mitogenomes. Plus indicate presence and dash indicate absence. *ARB*: *Gelidium arborescens*, *CRI*: *G. crinale* f. *luxurians*, *ELE*: *G. elegans*, *GAL*: *G. galapagense*, *ISA*: *G. isabelae*, *SCL*: *G. sclerophyllum*, *SIN*: *G. sinicola*, *VAG*: *G. vagum*, *MED*: *Pterocladia media*, *MEX*: *P. mexicana*, *MUS*: *P. musciformis*, *ROB*: *P. robusta*.

tRNAs	Anticodon	*ARB*	*CRI*	*ELE*	*GAL*	*ISA*	*SCL*	*SIN*	*VAG*	*MED*	*MEX*	*MUS*	*ROB*
trn-Asp	GTC	+	+	+	+	+	+	+	+	+	+	+	+
trn-Gly	TCC	-	+	-	-	-	-	-	-	-	+	-	+
trn-Gln	TTG	+	+	+	+	+	+	+	+	+	+	+	+
trn-Leu	TAA	+	+	+	+	+	+	+	+	+	+	+	+
trn-Leu	TAG	+	+	+	+	+	+	+	+	+	+	+	+
trn-Gly	GCC	+	+	+	+	+	+	+	+	+	+	+	+
trn-His	GTG	+	-	+	+	-	+	-	-	+	-	+	-
trn-Phe	GAA	+	+	+	+	+	+	+	+	+	+	+	+
trn-Ser	TGA	+	+	+	+	+	+	+	+	+	+	+	+
trn-Pro	TGG	+	+	+	+	+	+	+	+	+	+	+	+
trn-Cys	GCA	+	+	+	+	+	+	+	+	+	+	+	+
trn-Met	CAT	+	+	+	+	+	+	+	+	+	+	+	+
trn-Sec	TCA	+	+	+	+	+	+	+	+	+	+	+	+
trn-Ala	TGC	+	+	+	+	+	+	+	+	+	+	+	+
trn-Asn	GTT	+	+	+	+	+	+	+	+	+	+	+	+
trn-Val	TAC	+	+	+	+	+	+	+	+	+	+	+	+

trn-Arg	ACG	+	+	+	+	+	+	+	+	+	+	+	+
trn-Lys	TTT	+	+	+	+	+	+	+	+	+	+	+	+
trn-Glu	TTC	+	+	+	+	+	+	+	+	+	+	+	+
trn-Met	CAT	+	+	+	+	+	+	+	+	+	+	+	+

Table S4. A comparison of 23 protein-coding genes for 12 Gelidiales species. Length, variable sites, pairwise divergences, and Ka/Ks ratio. [a]Aligned length, [b]two species are merged with *P. capillacea*, [c]mean value, [†]barcoding region in *cox*1.

Gene	Length[a]	Variable site (%)	Pairwise divergences				Ka/Ks ratio[c]
			Gelidium	*Pterocladia*	*P. mexicana* vs *P. robusta*[b]	Total[c]	
*atp*6	768	320 (41.7)	8.3–18.0	7.7–18.4	0.8	18.4	0.0583
*atp*8	417	258 (61.9)	9.1–28.3	14.4–27.0	0.8	29.8	0.2884
*atp*9	231	54 (23.4)	2.2–10.8	3.5–9.1	0.4	9.9	0.0022
cob	1161	446 (38.4)	6.9–17.5	6.6–15.4	0.7	15.8	0.0494
COI-5P[†]	664	226 (34.0)	7.4–14.9	8.1–14.6	0.8	14.6	-
*cox*1	1599	544 (34.0)	7.0–15.3	8.1–13.9	0.4	14.3	0.0278
*cox*2	783	309 (39.5)	6.7–15.6	9.2–17.5	0.3	16.9	0.0509
*cox*3	819	320 (39.1)	6.6–15.1	7.4–14.9	0.4	16.3	0.0610
*nad*1	984	384 (39.0)	6.5–18.0	8.5–16.9	0.5	16.8	0.0439
*nad*2	1485	841 (56.6)	9.2–24.5	12.1–24.8	0.6	25.8	0.2020
*nad*3	366	158 (43.2)	5.7–17.5	10.9–17.8	1.1	19.0	0.0739
*nad*4	1476	683 (46.3)	7.6–18.8	9.9–19.9	0.7	19.6	0.0874
*nad*4L	306	111 (36.3)	5.2–14.4	6.2–15.0	0.3	14.8	0.0405
*nad*5	1998	981 (49.1)	9.0–20.2	10.3–21.0	0.8	22.1	0.1179
*nad*6	609	329 (54.0)	8.4–21.8	10.2–21.5	0.2	23.1	0.1494
*rpl*16	408	249 (61.0)	9.6–24.0	14.6–27.4	1.0	27.9	0.1238
*rps*3	729	442 (60.6)	10.2–30.1	14.4–30.3	0.9	32.5	0.3191
*rps*11	360	202 (54.7)	7.8–25.5	10.7–27.8	0.9	27.1	0.2459
*rps*12	390	174 (44.6)	6.3–19.7	10.7–22.4	0.3	21.2	0.0797
*sdh*2	762	360 (47.2)	8.6–19.0	11.5–23.8	0.9	21.0	0.1004
*sdh*3	393	265 (67.4)	11.5–29.9	14.4–30.4	0.3	33.3	0.3371
*sdh*D	243	152 (62.6)	8.2–24.7	11.1–28.8	0.4	27.5	0.3621
*sec*Y	798	566 (70.9)	12.9–32.5	16.5–35.0	0.7	35.6	0.3943
*ymf*39	543	362 (66.7)	9.6–27.3	13.1–26.0	0.0	29.4	0.2730

Table S5. Information about fresh specimens used in the present study.

Species	Voucher-code	Collection site; date	*cox*1	*rbc*L
Gelidium coulteri	UC2034155	Shaw's Cove, Orange County, CA, USA; 10.xii.2012	KX423456	-
	UC2026048	Corona del Mar, Orange County, CA, USA; 13.xii.2012	KX423457	-
	UC2026046	Corona del Mar, Orange County, CA, USA; 13.xii.2012	KX423458	-
	UC2025906	San Nicolas Island, Ventura County, CA, USA; 9.i.2013	KX423459	-
	UC2025904	San Nicolas Island, Ventura County, CA, USA; 9.i.2013	KX423460	-
	UC2025905	San Nicolas Island, Ventura County, CA, USA; 9.i.2013	KX423461	-
	UC2025907	San Nicolas Island, Ventura County, CA, USA; 13.xi.2012	KX423462	-
	CNU024853	Bajamar, Mexico; 24.ii.2010	KX423463	-
	CNU024854	Bajamar, Mexico; 24.ii.2010	KX423464	-
	CNU024855	Bajamar, Mexico; 24.ii.2010	KX423465	-
	CNU024859	Bajamar, Mexico; 24.ii.2010	KX423466	-
Gelidium millariana	CNU056286	Gordon's Bay, Sydney, Australia; 23.i.2014	KX423467	KX423484
	CNU056288	Gordon's Bay, Sydney, Australia; 23.i.2014	KX423468	KX423485
	CNU056289	Gordon's Bay, Sydney, Australia; 23.i.2014	KX423469	KX423486
	CNU056290	Gordon's Bay, Sydney, Australia; 23.i.2014	KX423470	-
Gelidium 'galapagense'	CNU053479	Doduri, Jeju, Korea; 17.vi.2008	KX423471	KX423487
	CNU053379	Geumneung, Jeju, Korea; 30.iii.2010	KX423472	-
	CNU054668	Geumneung, Jeju, Korea; 30.iii.2010	-	KX423488
	CNU052132	Gwideokri, Jeju, Korea; 19.x.2007	KX423473	KX423489
